# Inhibition of 3-mercaptopyruvate sulfurtransferase enhances CD8^+^ T-cell antitumor immunity

**DOI:** 10.1016/j.redox.2026.104373

**Published:** 2026-08-28

**Authors:** Muriel Urwyler, Marietta Margareta Korsos, Eloise Dupuychaffray, Nikita Markov, Kelly Ascenção, Maria Petrosino, Karim Zuhra, Darko Stojkov, Sara Barcos, Jérémie Tachet, Pragallabh Purwar, Montserrat Alvarez, Aurélien Pommier, Mohamed Z. Gad, Reham M. Abdel-Kader, Csaba Szabo, Carole Bourquin

**Affiliations:** a Institute of Pharmaceutical Sciences of Western Switzerland, University of Geneva, Switzerland; b Institute of Pharmacology, University of Bern, Switzerland; c Section of Pharmacology, Department of Oncology, Microbiology and Immunology, University of Fribourg, Switzerland; d Theodor Kocher Institute, University of Bern, Switzerland; e Biochemistry Department, Faculty of Pharmacy and Biotechnology, German University in Cairo (GUC), Cairo, Egypt; f Pharmacology and Toxicology Department, Faculty of Pharmacy and Biotechnology, German University in Cairo, Cairo, Egypt; g University of London at European Universities in Egypt (EUE), New Administrative Capital, Egypt; h Department of Anaesthesiology, Pharmacology, Intensive Care and Emergency Medicine, University of Geneva, Switzerland

**Keywords:** Hydrogen sulfide (H_2_S), 3-mercaptopyruvate sulfurtransferase (3-MST), Tumor immunotherapy, Immune checkpoint inhibition, Renal cell cancer

## Abstract

Hydrogen sulfide (H_2_S) is a redox-active gasotransmitter implicated in tumor progression and immune regulation. The enzyme 3-mercaptopyruvate sulfurtransferase (3-MST) is a key contributor to endogenous H_2_S and polysulfide production, but its role in tumor–immune interactions remains poorly defined. Here, we show that 3-MST is the most abundantly expressed H_2_S-synthesizing enzyme in human renal cell carcinoma cells (RCC) and that high 3-MST expression correlates with reduced patient survival. Pharmacological inhibition of 3-MST lowered intracellular H_2_S levels in Renca renal carcinoma cells, suppressed proliferation, induced apoptosis, disrupted cellular metabolism, and increased expression of immune-related genes and proteins. In immune cells, partial inhibition of 3-MST promoted T cell activation, as evidenced by increased CD69 expression on CD3^+^, CD4^+^, and CD8^+^ T cells. In contrast, complete inhibition of 3-MST, achieved by high concentrations of the inhibitor, modestly reduced CD8^+^ T cell proliferation. Functionally, 3-MST inhibition potentiated antigen-specific CD8^+^ T cell-mediated killing of tumor cells, an effect further amplified by PD-L1 blockade. These results establish 3-MST as a redox-sensitive metabolic driver of tumor growth and immune evasion in RCC and demonstrate that its inhibition can boost antitumor immune responses, offering a potential avenue for combination immunotherapy.

## Highlights

• 3-MST is the predominant H_2_S-producing enzyme in renal cell carcinoma.
• High 3-MST expression is associated with reduced survival in RCC patients.
• 3-MST inhibition disrupts tumor cell metabolism and induces apoptosis.
• 3-MST inhibition does not impact viability or activation of CD8^+^ T cells.
• 3-MST inhibition enhances CD8^+^ T-cell killing and PD-L1 blockade effects.

## Graphical abstract

**Figure:**
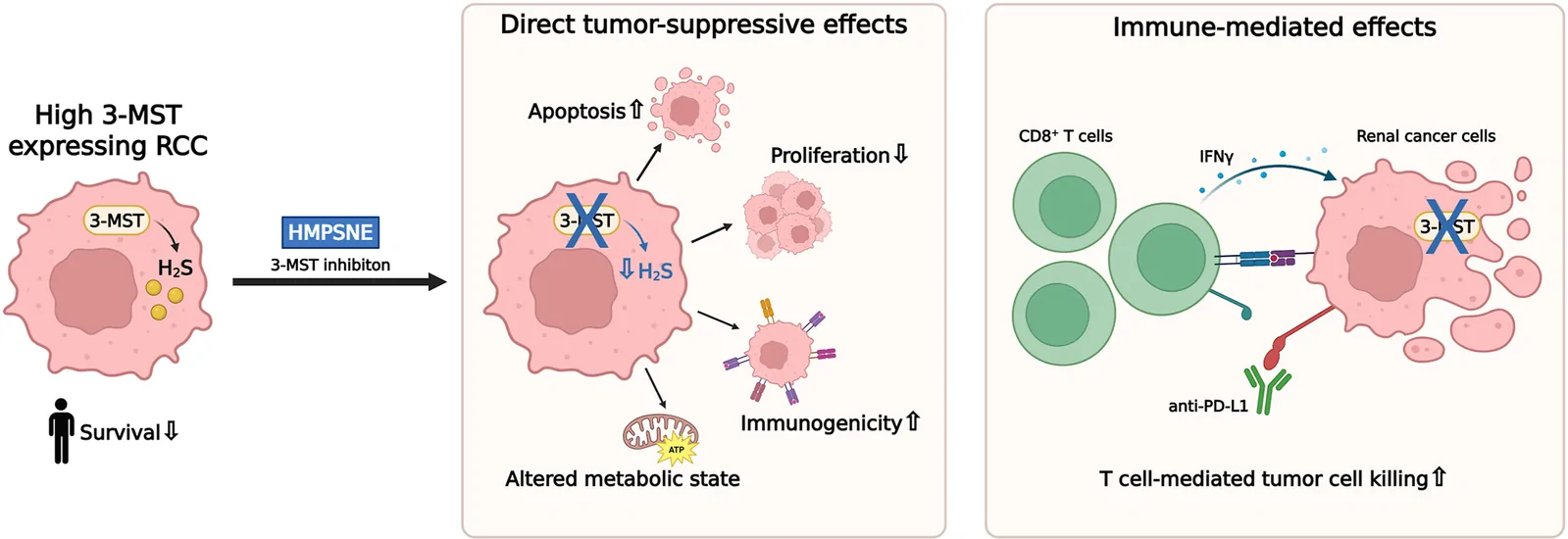

## 1 Introduction

Renal cell carcinoma (RCC) accounts for approximately 90% of all kidney cancers and remains a significant clinical challenge due to its resistance to conventional therapies [1,2]. Immunotherapy represents an important option for patients with RCC and is often used in combination with conventional treatments. The mainstay of immunotherapy in RCC are immune checkpoint inhibitors, which prevent immunosuppression of CD8^+^ T cells and restore their anticancer functionality [1]. Checkpoint inhibition has improved outcomes in many patients with advanced RCC, but therapeutic resistance and limited response rates highlight the need for novel strategies to sensitize these tumors to immune-mediated elimination [3].

Hydrogen sulfide (H_2_S) is an endogenously produced gaseous signaling molecule involved in a wide range of physiological and pathophysiological processes, including mitochondrial respiration, redox balance, and inflammation [4]. In the context of cancer, H_2_S has been implicated in supporting tumor growth and survival by promoting metabolic flexibility, angiogenesis, and resistance to oxidative stress [[5], [6], [7], [8]]. Beyond its effects on tumor cells, H_2_S also influences the immune response by regulating T cell activation, cytokine production, and the expression of immune checkpoint molecules and may thus impact antitumor immunity [9,10]. In mammalian cells, H_2_S is synthesized by three main enzymes: cystathionine β-synthase (CBS), cystathionine γ-lyase (CTH, also known as CSE), and 3-mercaptopyruvate sulfurtransferase (3-MST, encoded by *MPST*) [4]. While these enzymes have been extensively studied in cancer metabolism and inflammation [[11], [12], [13]], their precise roles — and that of 3-MST in particular — in antitumor immunity remain insufficiently characterized [14].

3-MST is partially localized to the mitochondria [15], where it catalyzes the conversion of 3-mercaptopyruvate into H_2_S, polysulfides, and other reactive sulfur species, thereby contributing to redox buffering and cellular antioxidant defenses [16,17]. Recent studies suggest that 3-MST contributes to the metabolic adaptability and survival of tumor cells, particularly under stress conditions such as hypoxia or oxidative challenge. However, its role in tumor immune evasion or immune modulation has not been investigated [18]. Interestingly, 3-MST activity is detected in many tissues, but the highest activity of 3-MST was measured in the kidney [14].

In this study, we examined the role of 3-MST in tumor metabolism, immunogenicity, and immune cell interactions using the murine Renca renal carcinoma model. We used 2-[(4-hydroxy-6-methylpyrimidin-2-yl)sulfanyl]-1-(naphthalen-1-yl)ethan-1 (HMPSNE), a selective pharmacological inhibitor of 3-MST, to assess how suppression of this enzyme affects tumor cell growth and CD8^+^ T cell-mediated antitumor responses. Our findings demonstrate that 3-MST inhibition impairs tumor cell viability, enhances the expression of immunoregulatory surface molecules and selected immune-related genes, and promotes antigen-specific cytotoxic T cell activity, particularly when combined with immune checkpoint inhibition. These results highlight 3-MST as a potential target for modulating tumor-immune interactions and improving immunotherapeutic strategies in patients with renal cancer.

## 2 Methods

### 2.1 Immunohistochemistry (IHC) analysis of H_2_S-synthesizing enzymes in normal kidney and RCC

**Fig. 1:**
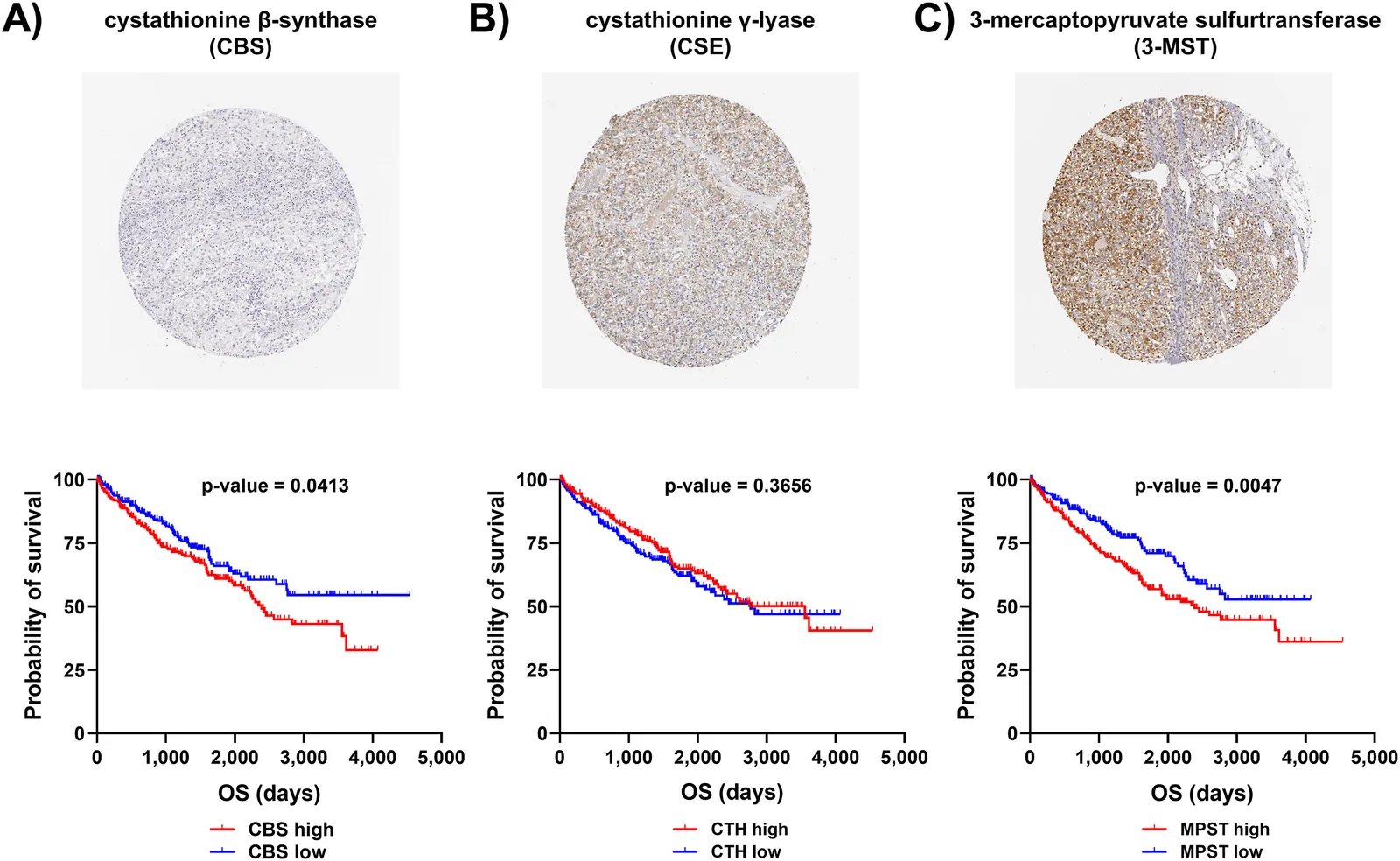
Expression of H_2_S synthesizing enzymes and correlation with survival in human RCC. **Top row:** Representative images of immunohistochemistry (IHC) staining of human renal cell adenocarcinoma (RCC) samples from the Human Protein Atlas showing expression of (**A**) cystathionine β-synthase (CBS), (**B**) cystathionine γ-lyase (CSE), and (**C**) 3-mercaptopyruvate sulfurtransferase (3-MST). **Bottom row:** Kaplan–Meier curves showing the overall survival (OS) for RCC patients stratified by high vs. low mRNA expression levels of (**A**) CBS, (**B**) CTH (encoding CSE), and (**C**) MPST (encoding 3-MST). Patients were dichotomized into high vs. low gene expression groups based on the median expression values as threshold. Data derived from The Cancer Genome Atlas (TCGA) Kidney Renal Clear Cell Carcinoma (KIRC) cohort (n = 533; low-expression group: n = 267, high-expression group: n = 266). Mantel-Cox test p-values are shown.

IHC data were obtained from the Human Protein Atlas (HPA, version 24; https://v24.proteinatlas.org) [19]. Images and expression annotations for both normal kidney and RCC samples were retrieved from the “Tissue” and “Pathology” sections of the HPA. Normal kidney data were used to describe baseline distribution of each enzyme in specific nephron segments and glomerular structures, while RCC data were analyzed for presence or absence of staining, staining intensity (not detected, low, medium, high), and proportion of tumor cells stained, as provided by the HPA (CBS, 3-MST: n = 12; CSE/CTH: n = 11). Representative high-resolution images were downloaded from the HPA for presentation in Fig. 1 and Fig. S1.

### 2.2 Transcriptomic analysis of TCGA data

Survival, immune scores and gene expression analyses were performed on the Cancer Genome Atlas Kidney Clear Cell Carcinoma (TCGA-KIRC) cohort (n = 533; https://www.cancer.gov/tcga). Survival analyses were performed using the UCSC Xena Browser [20] (https://xenabrowser.net). Patients were dichotomized into high and low expression groups based on the median cutoff values. To ensure consistency, only primary tumor samples (TCGA sample type code: 01) were retained for analysis. Duplicate entries and rare sample types, such as “additional new primary” tumors, were removed according to the UCSC Xena Browser's recommended preprocessing guidelines. Data was exported to GraphPad Prism software for figure design and statistical analysis. Immune scores were calculated using the Estimation of STromal and Immune cells in MAlignant Tumors using Expression data (ESTIMATE) algorithm [21]. Gene correlation were analyzed using the TIMER2.0 web platform [22].

### 2.3 Reagents

6-Methyl-2-((2-(naphthalen-1-yl)-2-oxoethyl)thio)pyrimidin-4(3H)-one (I3MT-3, HMPSNE, Otava Chemicals, CAS 459420-09-8) was first prepared as a 500 mM stock solution in DMSO, then further diluted 1:10 in DMSO. From this intermediate solution, a 5 mM working solution of HMPSNE was prepared in complete cell culture medium for experimental use. Similar dilution of DMSO into complete cell culture medium was used as vehicle control (0 μM HMPSNE).

### 2.4 Cancer cell lines

The Renca cell line (ATCC, CRL-2947) was genetically modified to express MHC Class I H2-K^b^ instead of the native H2-K^d^ (Renca H2-K^b^) and to express GFP (Renca H2-K^b^ GFP), as described previously [23]. Cells were cultured in RPMI-1640 medium (Gibco, #21875034) supplemented with 10% FBS (Capricorn Scientific, Germany), 100 U/mL penicillin/streptomycin (Gibco, #15140122), 1% non-essential amino acids (Gibco, #11140035), 1 mM sodium pyruvate (Gibco, #11360039), and 2 mM l-glutamine (Gibco, #25030024), (complete RPMI medium) at 37 °C in a 5% CO_2_ humidified atmosphere. Cells were seeded in 96-well plates (Greiner CellStar, #655180) and allowed to adhere for 24 h prior to HMPSNE treatment.

### 2.5 Quantification of H_2_S production

H_2_S produced by Renca cells was measured using the AzMC probe as previously described [24,25]. The day of the experiment, culture medium was replaced with HBSS buffer (Gibco, #14025-050) supplemented with the H_2_S-selective fluorescent probe 7-azido-4-methylcoumarin (AzMC; Sigma, #802409) 1 mM and d-glucose 4.5 g/l (Gibco, #A24940-01) as previously described [25], and further incubated for 1 h. Afterwards, cells were washed once in HBSS buffer supplemented with d-glucose 4.5 g/l and dye's specific fluorescence was visualized using a Leica DFC360 FX inverted microscope equipped with DAPI filter and images were captured with Leica Application Suite X software (Leica Biosystems, Germany) or a Biotek Cytation 5 imager (Agilent) equipped with DAPI cube filter (excitation 377/50 nm, emission 447/60 nm) and Gen5 software (Agilent, Santa Clara, CA, USA). Images were processed with Image J package (NIH, Bethesda, MD, USA) by applying a background subtraction followed by brightness contrast adjustment applying the same parameters for all images within the same experimental day. Quantitative analysis of the AzMC fluorescence signal was performed by using the Spectramax id5 (Molecular Devices, San Jose, CA, USA) (excitation 365 nm, emission 450 nm) and data graphed with GraphPad Prism 8 (GraphPad Software Inc.; San Diego, California, USA).

### 2.6 Quantification of polysulfide production

The day of the experiment, cells were washed once with serum-free medium and then incubated for 15 min at 37 °C and 5% CO_2_ with the polysulfide selective fluorescent probe SSP4 (Dojindo Molecular Technologies, #SB10) at 20 μM final concentration, prepared in serum-free medium and supplemented with 0.5 mM Cetyltrimethylammonium bromide (CTAB). Afterwards, cells were washed three times in PBS buffer and dye's specific fluorescence was visualized using a Biotek Cytation 5 imager (Agilent) equipped with GFP cube filter (excitation 469/35 nm, emission 525/39 nm) and Gen5 software (Agilent, Santa Clara, CA, USA). Images were processed with Image J package (NIH, Bethesda, MD, USA) by applying a background subtraction followed by brightness contrast adjustment applying the same parameters for all images within the same experimental day. Quantitative analysis of the SSP4 fluorescence signal was performed by using the Spectramax id5 (Molecular Devices, San Jose, CA, USA) (excitation 482 nm, emission 515 nm) and data graphed with GraphPad Prism 8 (GraphPad Software Inc.; San Diego, California, USA).

### 2.7 Confocal laser scanning microscopy

To assess 3-MST intracellular protein expression, Renca cells, treated either with 30 μM of HMPSNE or vehicle, were seeded on untreated German glass coverslips (6 mm in thickness and 12 mm in diameter, Karl Hecht GmbH & Co. KG “Assistent”, Sondheim/Rhön, Germany), which had previously been washed with acetone, ethanol, and ddH_2_O and baked in an oven. After 48 h, attached cells were stained with MitoTracker Orange (1 μM), prior to fixation. Cells were fixed with 4% paraformaldehyde (ThermoFisher Scientific) for 10 min. After washing with PBS pH 7.4, samples were permeabilized with 0.5% Triton X-100 (Calbiochem, Merck Millipore) in PBS, pH 7.4 for 10 min and blocked in blocking buffer (7.5% BSA, 30% normal goat sera (Santa Cruz Biotechnology), in PBS, pH 7.4) for 1 h at room temperature. Rabbit anti-MST antibody (#ab154514) was diluted 1:100 in blocking buffer and cells were stained overnight at 4 °C. Cells were washed three times in PBS, pH 7.4 with 0.025% Triton X-100, and then further incubated with the secondary antibody Alexa-488 goat anti–rabbit IgG H&L (1:1000 in 7.5% BSA with 0.025% Triton X-100; ThermoFisher Scientific) for 1 h at room temperature. Samples were extensively washed with PBS pH 7.4 with 0.025% Triton X-100, and nuclei were counterstained with 1 μg/ml Hoechst 33342 (ThermoFisher Scientific) for 10 min at room temperature in the dark. Cells were subsequently washed three times in PBS, pH 7.4, and mounted in ProLong Gold mounting medium (ThermoFisher Scientific). Slides were examined, and images were acquired by an LSM 910 (Carl Zeiss Micro Imaging, Jena, Germany) with a 63×/1.40 oil differential interference contrast objective, followed by analysis with IMARIS software as previously reported [26].

### 2.8 Western blotting

Cells were lysed in RIPA buffer with a protease inhibitor cocktail (Merck, #P8340), and 1 mM PMSF. Extracted proteins were denatured with 100 mM dithiothreitol and LDS Sample Buffer (Abcam, #ab119196). Proteins were separated on SERVAGel TG PRiME gels (SERVA Electrophoresis) and transferred to Immobilon-P PVDF membranes (Merck Millipore, #IPVH00010). Membranes were blocked with 5% non-fat milk in TBST (0.1% Tween 20, 20 mM Tris, 150 mM NaCl, pH 7.6) for 1 h and incubated overnight at 4 °C with primary antibodies diluted in 5% BSA in TBST: MST antibody (Abcam, #ab154514; 1:1000), Lamin A/C antibody (Cell Signaling, #2032, 1:1000). After washing, membranes were incubated with HRP-conjugated secondary antibodies (GE Healthcare Life Sciences) and developed using Immobilon Forte Western HRP substrate (Merck Millipore, #WBLUF0500) on an Odyssey Fc Imaging System (LI-COR Biosciences).

### 2.9 Renca cell proliferation

Renca cell proliferation was monitored in real-time using the Incucyte Live-Cell Imaging System (Sartorius). Renca-H2-Kb GFP^+^ cells were seeded in a 96-well plate at a density of 5 × 10^3^ cells/well and cell proliferation was quantified by measuring GFP signal over time using the Incucyte's integrated software (Sartorius).

### 2.10 Flow cytometry

Flow cytometry was performed using a NovoCyte 3000 (ACEA Biosciences). Single-cell suspensions from tissues or cultures were stained with Zombie Violet™ (BioLegend, #423114) to exclude dead cells. Cells were incubated with anti-CD16/32 (BioLegend, #101320) and fluorochrome-conjugated antibodies (Supplementary Table 1) for 30 min at 4 °C. Staining was performed in FACS buffer (PBS + 0.5% BSA + 2% EDTA). Apoptosis was assessed using Annexin V-Pacific Blue (BioLegend, #640917) and propidium iodide (Sigma, #P4170) per protocol. Data were analyzed using FlowJo™ v10.10.0 (FlowJo LLC). FACS panels and gating strategies are detailed in the Supplementary Materials.

### 2.11 siRNA-mediated silencing of 3-MST in Renca cells

Renca cells were transfected using HiPerFect Transfection Reagent (Qiagen, #301705) with either non-targeting control siRNA (Dharmacon, #D-001206-14-05; siCTRL cells) or MPST-targeting siRNA (Qiagen, #1027416; siMPST cells). For each well, 20 pmol siRNA (in 10 μL) and 10 μL HiPerFect reagent were mixed with 90 μL of serum-free RPMI-1640 medium, briefly vortexed, and incubated for 10 min at room temperature. Subsequently, 100 μL of the resulting transfection mixture was then added dropwise to the corresponding well of a 6-well plate containing approximately 2 × 10^4^ Renca cells in 1 mL of complete medium. After 48 h, the medium was replaced with 2 mL of fresh complete medium. Cells were analyzed 72 h after transfection, when maximal MPST knockdown was observed.

### 2.12 Analysis of bioenergetic profiles using Seahorse XF96

Renca cells were seeded in 96-well Seahorse XF poly-l-lysine pre-coated plates (0.01% w/v in ddH_2_O, 1 h at room temperature) at a density of 9 × 10^4^ cells per well in complete Seahorse XF DMEM media (Agilent, #103575-100) containing 11 mM glucose (Agilent, #103577-100), 2 mM l-glutamine (Agilent, #103579-100), and 1 mM sodium pyruvate (Agilent, #103578-100). Plates were then incubated for 1 h at 37 °C in a non-CO_2_ incubator to allow temperature and pH equilibration. Stock solutions of oligomycin, FCCP, rotenone, antimycin A, and 2-deoxy-d-glucose (all from Sigma-Aldrich) were prepared in the same assay medium and loaded into the injection ports of the Seahorse cartridge. Oxygen consumption rate (OCR) and extracellular acidification rate (ECAR) were measured under basal conditions and after sequential injections of 2 μM oligomycin, 3 μM FCCP (selected after titration), 2 μM rotenone + 2 μM antimycin A, and 50 mM 2-deoxy-d-glucose using the Seahorse XFe96 Analyzer (Agilent). This design of the assay allowed calculation of multiple parameters related to glycolysis and mitochondrial respiration including basal mitochondrial respiration (ΔOCR between the steady state and after rotenone + antimycin A injection), ATP-linked respiration (ΔOCR between basal mitochondrial respiration and after oligomycin injection), glycolysis (ΔECAR between the steady state and after 2-DG injection), spare glycolytic capacity (ΔECAR between the steady state and after oligomycin injection). The OCR/ECAR ratio was calculated by dividing basal mitochondrial respiration by glycolysis.

### 2.13 Measurement of mitochondrial membrane potential, biomass and ROS

Mitochondrial membrane potential (MMP) and biomass were measured by flow cytometry as previously described [27]. In brief, Renca cells were trypsinised, washed, and resuspended at 1 × 10^6^ cells/mL in complete media containing either 200 nM of MitoTracker™ Green FM (Thermo Fisher Scientific, #M7514) for mitochondrial biomass, 3 μM JC-1 (Thermo Fisher Scientific, #T3168) for MPP, or 10 μM Dihydrorhodamine 123 (Merck, #309825-5 MG) for reactive oxygen species (ROS), for 30 min at 37 °C and then acquired with BD FACSLyric™ flow cytometer (BD Biosciences).

### 2.14 RNA sequencing and transcriptomics analysis

Renca cells treated with 50 μM HMPSNE or vehicle for 72 h, Renca cells transfected with siCTRL or siMPST for 72 h, and CD8^+^ T cells treated with 50 μM HMPSNE or vehicle and activated with Dynabeads (anti-CD3/CD28) for 24 h, were subjected to transcriptomics analysis by RNA sequencing. Total RNA was isolated using the RNeasy Plus Mini Kit (Qiagen, #74134). Library preparation and sequencing were performed by Novogene. Poly(A)-enriched RNA libraries were generated by mRNA capture followed by reverse transcription. Libraries were sequenced using a 150 bp paired-end strategy on a NovaSeq X Plus platform, yielding an average of approximately 20 million paired-end reads per sample. Adapter sequences and low-quality bases were removed using Fastp. Clean reads were aligned to the mouse reference genome (GRCm39) using STAR, and gene-level read counts were generated with FeatureCounts. Transcript abundance was estimated as transcripts per million (TPM) and fragments per kilobase of transcript per million mapped reads (FPKM) using StringTie. Differential gene expression analysis was performed using DESeq2 and edgeR. RNA-Seq data processing and downstream analyses were performed using the Galaxy platform [28], the European Galaxy server, the published RNA-Seq workflow (RNA-Seq for Paired-end FASTQs by iwc, release v1.1), iDEP, and ShinyGO [29,30].

### 2.15 Mouse splenocytes

To generate single-cell suspensions, spleens from 6- to 16-week-old female C57BL/6J or OT-I (C57BL/6-Tg(TcraTcrb)1100Mjb/Crl) mice were passed through a 40 μm filter and treated with Pharm Lyse™ red blood cell lysis buffer (BD, #555899). CD8a^+^ T cells were isolated from single-cell suspensions using a magnetic negative selection kit (Miltenyi Biotec, #130-104-075), following the manufacturer's protocol. Isolation efficiency was validated by flow cytometry. Splenocytes were activated by addition of the TLR7 agonist R848 (Invivogen, #tlrl-r848-5) at a concentration of 25 ng/mL at the same time as HMPSNE.

### 2.16 Generation of Bone Marrow-Derived Dendritic Cells (BMDC)

BMDC were generated from 6- to 16-week-old female C57BL/6J mice as described [31]. Bone marrow was flushed from femurs and tibiae with PBS and treated with Pharm Lyse buffer. After filtration through a 40-μm strainer, cells were seeded in 6-well plates at 2.5 × 10^6^ cells/mL in complete RPMI medium with 20 ng/mL GM-CSF (Peprotech, #315-03) and cultured in a 37 °C, 5% CO_2_ humidified environment. The medium was refreshed on days 2 and 3 after isolation. On day 6, BMDC were harvested and plated into 96-well plates at 1-1.5 × 10^5^ cells/well. Purity was verified by flow cytometry.

### 2.17 Quantification of cellular ATP levels

Renca cells (5 × 10^3^ cells/well) were seeded and allowed to adhere for 24 h prior to HMPSNE treatment. Splenocytes and BMDCs (1 × 10^5^ cells/well) were seeded in 96-well plates and treated with HMPSNE immediately after plating. Cellular ATP levels, which indicates the presence of metabolically active cells, were measured after 24 and 48 h by CellTiter-Glo® (Promega, #G7572) according to the manufacturer's instructions using a Clariostar Plus plate reader.

### 2.18 CD8^+^ T-cell proliferation

CD8^+^ T cells isolated from OT-I mice were resuspended at 1 × 10^7^ cells/mL in PBS with 0.5 μM CFSE (BioLegend, #423801) for 10 min at room temperature. Cells were plated in complete RPMI medium supplemented with 50 μM 2-mercaptoethanol (Gibco, #31350010) at 1 × 10^5^ cells/well in 96-well plates. CD8^+^ T cells were activated with mouse IL-2 (30 U/mL; Gibco, #210-12-10UG) and CD3/CD28 Dynabeads (1:1 bead-to-cell ratio, Gibco, #1145D) during exposure to HMPSNE. After 24 and 48 h, beads were removed using a DynaMag magnet (Invitrogen), and cells were analyzed by flow cytometry. Cell culture supernatants were collected after 24 h, and IFNγ production was measured by ELISA assay (BioLegend, #430815) following manufacturer's recommendations.

### 2.19 CD8^+^ T cell-mediated anti-tumor cytotoxicity

Renca-H2-Kb GFP^+^ cells (1 × 10^3^ cells/well) were seeded in 96-well plates, allowed to adhere for 24 h, then treated with HMPSNE. Renca cells were pulsed with 2 μg/mL SIINFEKL peptide (InvivoGen, #vac-sin) for 4 h at 37 °C. OT-I CD8^+^ T cells (5 × 10^3^ cells/well) were added. Renca cell proliferation was monitored by measuring GFP intensity using the Incucyte® Live-Cell Analysis System (Sartorius). Anti-PD-L1 antibody (10 μg/mL; InvivoGen, #pdl1-mab15-1) was added where indicated. The same procedure was followed for the experiments using siRNA-treated Renca cells, with cell seeding performed 48 h after silencing.

### 2.20 Statistical analysis

Statistical analyses were performed using GraphPad Prism. Data are shown as mean ± standard deviation (SD) of biological replicates unless indicated otherwise. Statistical significance was assessed using one-way or two-way ANOVA with Dunnett's, Tukey's, Šidák's, or Bonferroni's multiple comparison tests as indicated. A p-value ≤ 0.05 was considered significant.

#### 2.20.1 Ethics statement

No new human samples were collected for this study, and all analyses for human tissues were performed on de-identified, previously published datasets. Animals were purchased from Charles River Laboratories and maintained under specific pathogen-free conditions at the Centre Médical Universitaire, University of Geneva, and at the Central Animal Facility, University of Bern. All animal experiments were approved by the veterinary authorities of the Canton of Geneva (Geneva, Switzerland) and the Canton of Bern (Bern, Switzerland) under the licenses GE182, BE34a/2023, and BE19/2025, in accordance with the Swiss federal regulations.

## 3 Results

### 3.1 3-MST is highly expressed in human renal cell cancer and correlates with poor prognosis

To assess the expression of H_2_S-synthesizing enzymes in human renal cell cancer (RCC), we examined immunohistochemistry (IHC) images from the Human Protein Atlas (HPA) [32]. In healthy kidney tissue, cystathionine β-synthase (CBS) was not detectable, whereas cystathionine γ-lyase (CSE) was expressed at high levels in proximal tubules and at lower levels in distal tubules but was not detectable in glomeruli (representative images in Fig. S1A). The staining pattern for 3-mercaptopyruvate sulfurtransferase (3-MST) showed strong positivity in the epithelial cells of the proximal tubules, whereas the distal tubules showed weak staining. Vascular structures were negative. In the glomerulus, the parietal epithelial cells of the Bowman's capsule were strongly positive. In renal cell adenocarcinoma, which arises from cells of the proximal tubule, CBS was not detected in any of the HPA samples (n = 12) (representative image in Fig. 1A–top row). CSE was not detected in 6 of 11 samples and detected at low (4/11) or medium (1/11) levels in the others (Fig. 1B–top row). In contrast, 3-MST was detected at low (6/12) to medium (2/12) levels in the majority of patients, clearly staining over 25% of tumor cells in the majority of the samples analyzed (Fig. 1C–top row).

We next examined the association between expression of H_2_S-synthesizing enzymes and patient survival using mRNA expression data from The Cancer Genome Atlas (TCGA) Kidney Renal Clear Cell Carcinoma (KIRC) cohort (n = 533). Patients were stratified into high and low expressers based on median transcript levels of *MPST* (encoding 3-MST), *CBS*, or *CTH* (encoding CSE). Kaplan-Meier survival analysis showed that low *CBS* expression was associated with improved survival (Fig. 1A, bottom row), whereas no difference was observed between high and low *CTH* expression groups (Fig. 1B, bottom row). In contrast, low *MPST* expression was strongly associated with improved overall survival (p = 0.0047), suggesting a potential role for 3-MST in promoting tumor aggressiveness (Fig. 1C, bottom row). Interestingly, patients with low expression of both *MPST* and *CBS* had significantly better overall survival than those in the other groups, suggesting a synergistic effect of the two enzymes (Fig. S1B). Additionally, we computationally assessed immune-cell infiltration in RCC samples according to *MPST* and *CBS* expression levels, using the ESTIMATE algorithm to calculate immune scores [21] and the TIMER2.0 platform to analyze correlations with immune-related gene expression [22]. Although total immune cell infiltration did not differ between groups (Fig. S1C), *MPST* expression was correlated with several immune genes encoding activation markers (CD69), costimulatory molecules (CD70, CD80, CD86), immunosuppressive proteins (Galectin-9, CD155), and immune checkpoints (PD-1, PD-L1) (Fig. S1D). In contrast, *CBS* expression did not correlate with these genes, except for LGLS3, which encodes the immunosuppressive protein Galectin-3. Collectively, these findings suggest that, among the three major H_2_S-producing enzymes, 3-MST is the most highly expressed in human RCC and that its elevated expression may correlate with worse clinical outcomes. To explore the functional relevance of 3-MST in renal cancer, we next investigated the effects of its pharmacological inhibition in renal cancer cells.

### 3.2 Pharmacological inhibition of 3-MST reduces intracellular H_2_S in renal cancer cells

**Fig. 2:**
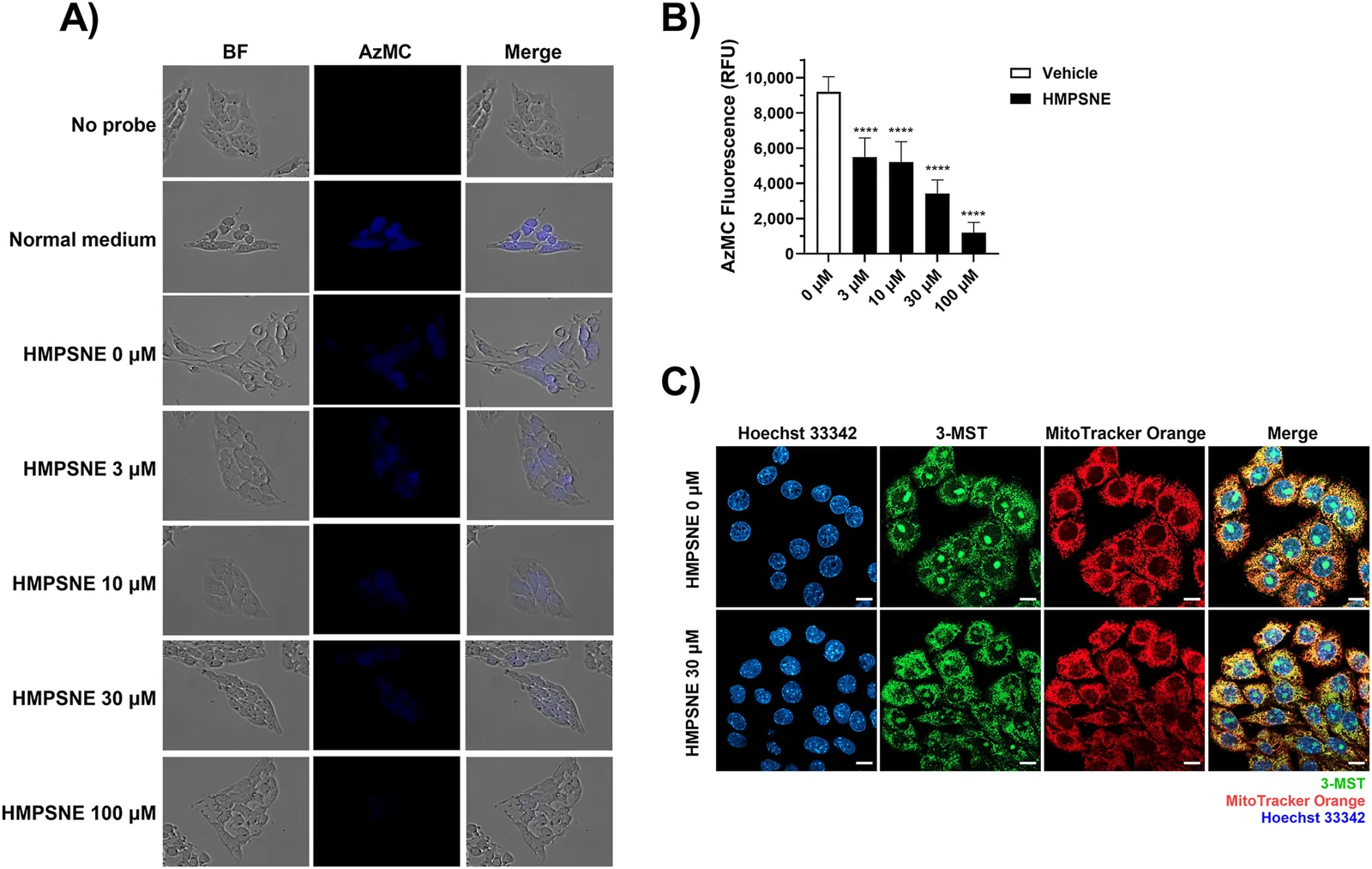
**H_2_S production and 3-MST localization in renal cancer cells following pharmacological inhibition of 3-MST.** (**A-B**) Cellular H_2_S content in Renca cells treated with increasing concentrations of HMPSNE for 48 h, quantified using the AzMC (7-azido-4-methylcoumarin) probe. (**A**) Representative bright-field (BF) and AzMC fluorescence images of cells. (**B**) Quantification of AzMC fluorescence signal across HMPSNE concentrations. Data represent the mean ± SD from six independent experiments. Statistical analysis was performed using one-way ANOVA with Dunnett's multiple comparisons test. ****p < 0.0001 vs. vehicle control. (**C**) 3-MST subcellular localization in Renca cells treated with HMPSNE (30 μM) or vehicle control, assessed by confocal microscopy. 3-MST was immunostained using an anti-3-MST antibody and a secondary anti-rabbit Alexa Fluor 488 antibody (green). Nuclei were stained with 33342 Hoechst (blue). Mitochondria were labelled with MitoTracker Orange (red). In the merged images, 3-MST is shown in green and yellow color. Scale bars: 10 μm.

**Fig. 3:**
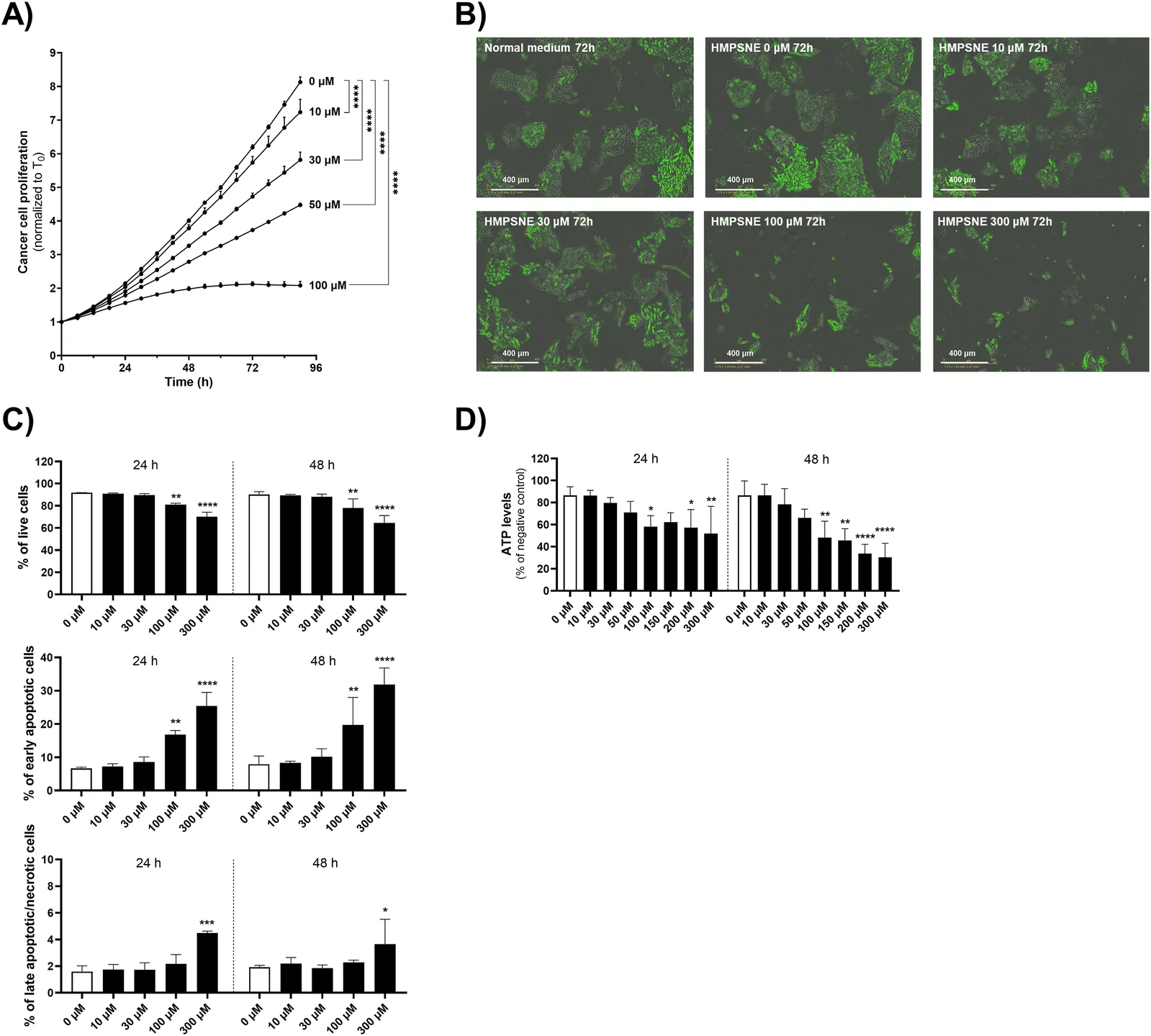
**Effect of 3-MST inhibition on renal cancer cell proliferation and viability.** Renca-H2-Kb GFP^+^ cells were treated with increasing concentrations of HMPSNE. (**A**) Live-cell imaging expressed as GFP signal normalized to the starting point (T_0_ = 1) for each well. Data are depicted as the mean ± SD from one experiment. (**B**) Representative images of cells after 72 h of HMPSNE treatment. Images were acquired using the Incucyte® Live-Cell Analysis System (20× objective). (**C**) Percentage of live cells (Annexin V^−^, PI^−^), early apoptotic cells (Annexin V^+^, PI^−^), late apoptotic/necrotic cells (Annexin V^−/+^, PI^+^) measured by flow cytometry. Data are depicted as the mean ± SD. (**D**) Percentage of metabolically active cells at 24 and 48 h assessed by quantifying cellular ATP levels using the CellTiter-Glo assay. Data are depicted as the mean ± SD from three independent experiments and normalized to negative control (untreated cells). Statistical analysis was performed using two-way ANOVA with Dunnett's multiple comparisons test. ****p < 0.0001; ***p < 0.001; **p < 0.01; *p < 0.05 vs. vehicle control.

First, we examined the impact of 3-MST inhibition on endogenous H_2_S production in renal cancer cells by quantifying intracellular H_2_S levels in murine Renca renal carcinoma cells treated with the selective 3-MST inhibitor HMPSNE. Fluorescence from the H_2_S-sensitive AzMC probe was detected in untreated cells and decreased upon treatment with increasing concentrations of HMPSNE (Fig. 2A). Quantification of the AzMC signal confirmed a dose-dependent reduction in fluorescence, indicating reduced intracellular H_2_S levels (Fig. 2B). Consistent with these findings, fluorescence imaging using the polysulfide-selective probe SSP4 showed a dose-dependent decrease in signal following HMPSNE treatment, indicating reduced polysulfide levels (Fig. S2A and B). As seen in Figs. 2C and 3-MST showed a predominant mitochondrial localization, with additional staining in structures resembling nuclear pseudoinclusions—cytoplasmic invaginations into the nucleus in cells with high proliferative and mitotic activity [33]. Importantly, HMPSNE did not impact 3-MST mitochondrial localization nor its overall protein abundance (Fig. 2C and Figure S2C and D). These results confirm that HMPSNE effectively suppresses H_2_S production in Renca cells and validate its utility for functional studies of 3-MST inhibition in renal cancer cells.

### 3.3 3-MST inhibition reduces proliferation and induces apoptosis in Renca cells

To investigate the functional impact of 3-MST inhibition on cancer cell proliferation, GFP-positive Renca cells (Renca H2-Kb GFP^+^) were treated with HMPSNE at concentrations ranging from 10 to 300 μM. Live-cell imaging revealed a concentration-dependent reduction in proliferation (Fig. 3A and B). The decrease in proliferation was accompanied by a dose-dependent increase in apoptotic cells at both 24 and 48 h post-treatment (Fig. 3C and Fig. S3A). Consistently, a reduction in the percentage of metabolically active cells, as assessed by quantification of cellular ATP levels, was observed from 100 μM HMPSNE (Fig. 3D). These findings demonstrate that pharmacological inhibition of 3-MST suppresses the proliferation of renal cancer cells.

### 3.4 3-MST inhibition disrupts the metabolic state of Renca cells

**Fig. 4:**
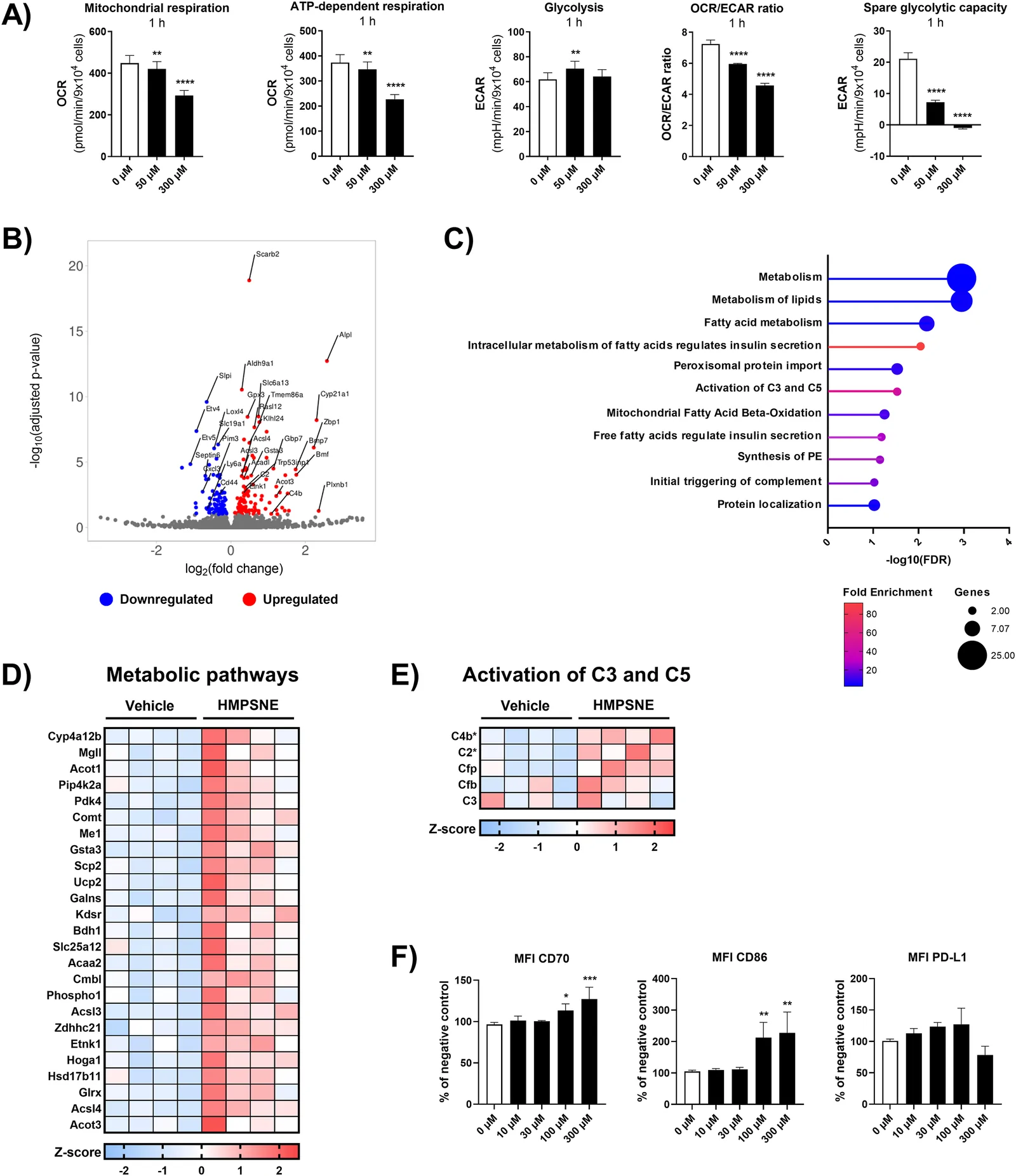
**Effect of 3-MST inhibition on metabolic state and gene expression of renal cancer cells.** Renca cells were treated with HMPSNE (50 or 300 μM) or vehicle control. (**A**) Bioenergetic profiling measured by extracellular flux analysis after 1 h of treatment (OCR: Oxygen Consumption Rate; ECAR: Extracellular Acidification Rate). Data are depicted as the mean ± SD (n = 3). Statistical analysis was performed using one-way ANOVA with Šidák's multiple comparisons test. ****p < 0.0001; **p < 0.01 vs. vehicle control. (**B-E**) Gene expression profile was analyzed by RNA-sequencing after 72 h of treatment (n = 4). (**B**) Volcano plots of the differentially expressed genes (DEG). Red circles and blue circles represent genes upregulated or downregulated, respectively, in HMPSNE-treated cells compared with vehicle-treated cells. (**C**) Enrichment analysis using the Reactome database on the DEG upregulated in HMPSNE-treated cells. (**D-E**) Heatmap of (**D**) the DEG from the “Metabolism” signature, and (**E**) the two DEG (*) and other genes from the “Activation of C3 and C5” signature. Data represent TPM values of four replicates and are displayed as Z-scores. (**F**) Expression of CD70, CD86, and PD-L1 determined by flow cytometry (MFI: mean fluorescence intensity). Data are depicted as the mean ± SD from three independent experiments and normalized to negative control (untreated cells). Statistical analysis was performed using one-way ANOVA with Dunnett's multiple comparisons test. ***p < 0.001; **p < 0.01 vs. vehicle control.

Since H_2_S contributes to mitochondria homeostasis, we next investigated whether 3-MST inhibition alters cellular bioenergetics. Acute HMPSNE treatment rapidly disrupted the metabolic state of Renca cells, as shown by a dose-dependent reduction in the OCR/ECAR ratio after 1 h of treatment, primarily driven by a decrease in mitochondrial and ATP-dependent respiration (Fig. 4A). Renca cells appeared to partially compensate for this early impairment: after 72 h of treatment, their respiration rate was no longer reduced, although their spare glycolytic capacity remained significantly impaired (Fig. S4A). At this time point, HMPSNE did not affect mitochondrial biomass, reactive oxygen species (ROS) production, or mitochondrial membrane potential (Fig. S4B–D). RNA-sequencing analysis provided further evidence of metabolic reprogramming, revealing the upregulation of a set of genes involved in lipid metabolism in HMPSNE-treated cells after 72 h of treatment (Fig. 4B–D). These changes may reflect a compensatory increase in fatty-acid catabolism in response to the initial reduction in mitochondrial respiration observed in HMPSNE-treated cells. These findings suggest that Renca cells partially adapt to HMPSNE-induced mitochondrial dysfunction, although the persistence of metabolic alterations indicate that this adaptation remains incomplete.

In parallel, we used an independent, siRNA-based approach to reduce H_2_S synthesis by targeting *Mpst*, the gene encoding 3-MST, in Renca cells. *Mpst* silencing markedly reduced its expression without inducing strong toxicity, as shown by the modest reduction in cell number (Fig. S5A–D), and significantly decreased H_2_S and polysulfide production (Fig. S5E and F). Consistent with the effects observed after 72 h of HMPSNE treatment, *Mpst* silencing did not alter the measured bioenergetic parameters (Fig. S5G). However, it significantly increased ROS production and mitochondrial membrane potential without concomitant increase in oxidative phosphorylation, suggesting impaired mitochondrial homeostasis and the potential accumulation of dysfunctional mitochondria in *Mpst*-silenced (siMPST) Renca cells (Fig. S5H–J). Collectively, these findings indicate that 3-MST inhibition negatively affects mitochondrial homeostasis and reduces the metabolic resilience of renal cancer cells.

### 3.5 3-MST inhibition enhances the immunogenic phenotype of Renca cells

Interestingly, 3-MST inhibition also altered the immunogenic phenotype of Renca cells. Enrichment analysis revealed the upregulation of genes associated with complement activation in HMPSNE-treated cells (Fig. 4E) and of genes involved in antigen processing and presentation pathways in siMPST cells (Fig. S5K and L). In addition, flow cytometry analysis showed increased surface expression of CD70 and CD86 at 100 and 300 μM HMPSNE, but no change in PD-L1 expression (Fig. 4F and Fig. S3B). These molecules play an important role in immune cell interactions and activation. CD70 and CD86 contribute to the activation and maintenance of T cell responses [34,35], whereas PD-L1 inhibits T-cell responses through immune checkpoint signaling [36]. No significant changes were observed in the expression of the immunosuppressive molecules CD155 and Galectin-9, which are frequently overexpressed in cancers [37,38], or in MHC class I expression (data not shown). Together, these findings indicate that 3-MST inhibition not only affects Renca cell proliferation and metabolism but also enhances the expression of immunostimulatory molecules and immune-related genes, potentially increasing tumor cell visibility to the immune system. We therefore next examined whether 3-MST inhibition also directly influences immune cell function.

### 3.6 3-MST inhibition has limited cytotoxicity on immune cells and supports T-cell activation

**Fig. 5:**
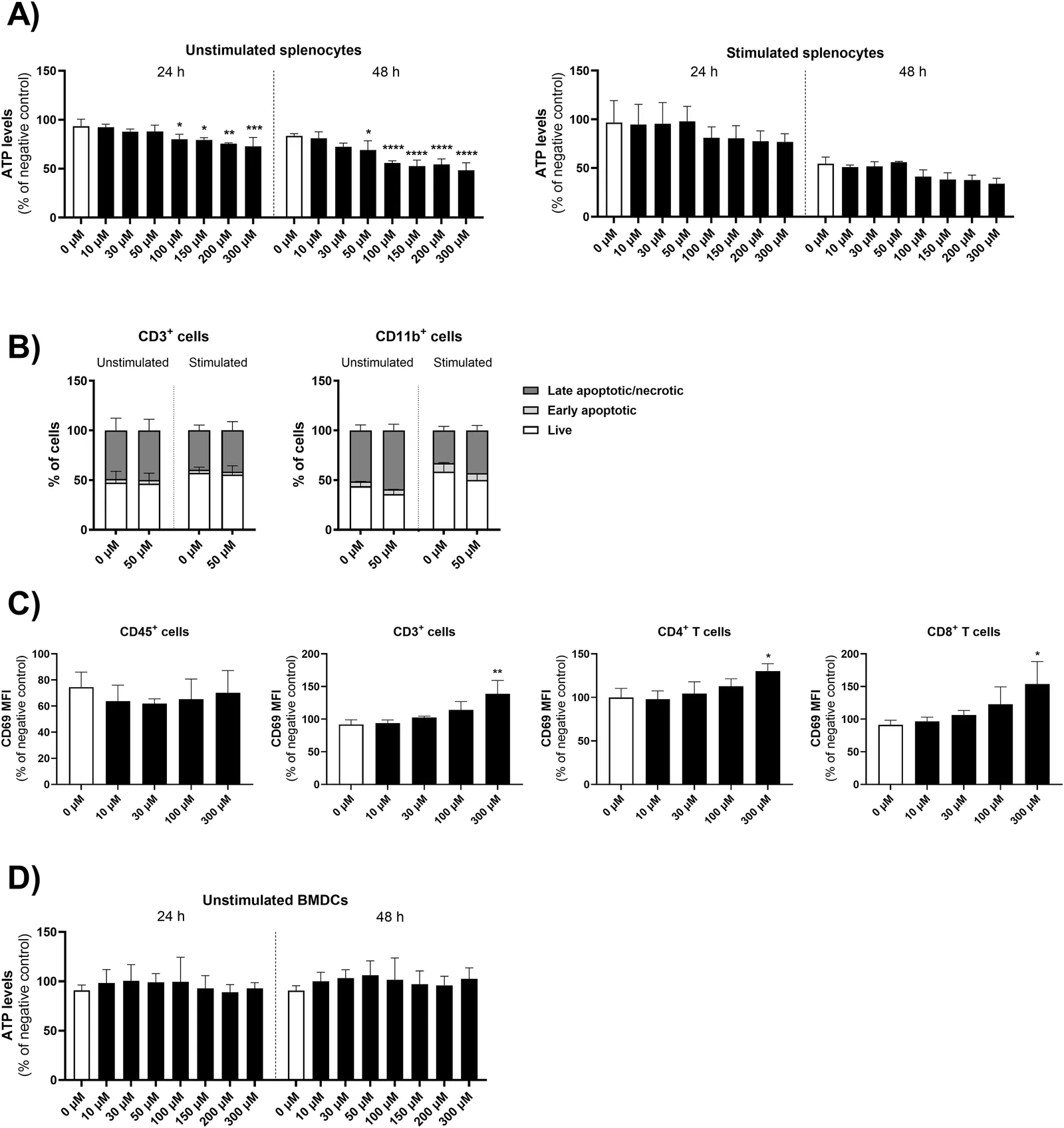
**Effect of 3-MST inhibition on viability and activation of immune cells.** (**A-B**) Mouse splenocytes were either left unstimulated or stimulated with R848 and treated at the same time with various concentrations of HMPSNE. (**A**) Percentage of metabolically active cells at 24 and 48 h assessed by quantifying cellular ATP levels using the CellTiter-Glo assay. (**B**) Percentage of live cells (Annexin V^−^, PI^−^), early apoptotic cells (Annexin V^+^, PI^−^), and late apoptotic/necrotic cells (Annexin V^−/+^, PI^+^) measured by flow cytometry in T cells (CD3^+^) and myeloid cells (CD11b^+^). (**C**) Flow cytometric analysis of CD69 expression on R848-activated CD45^+^ splenocytes, total T cells (CD3^+^), and T-cell subtypes: helper (CD4^+^) and cytotoxic (CD8^+^), (MFI: mean fluorescence intensity). (**D**) Metabolic activity of unstimulated bone marrow-derived dendritic cells (BMDC) was assessed at 24 and 48 h by quantifying cellular ATP levels using the CellTiter-Glo assay. Data represent the mean ± SD from three independent experiments and are normalized to negative control (untreated cells). Statistical analysis was performed using two-way ANOVA (A, D) and one-way ANOVA (C) with Dunnett's multiple comparisons test. ****p < 0.0001; ***p < 0.001; **p < 0.01; *p < 0.05 vs. vehicle control.

To evaluate the effects of 3-MST inhibition on the viability and functional activation of immune cells, mouse splenocytes were treated with HMPSNE at concentrations ranging from 10 to 300 μM. Murine splenocytes are a heterogeneous population of immune cells composed of B and T lymphocytes as well as myeloid cells such as monocytes and dendritic cells [39]. Percentage of metabolically active splenocytes, as assessed by quantification of cellular ATP levels, was slightly reduced in presence of high concentrations of HMPSNE after 24 and 48 h (Fig. 5A, left panel). A trend towards a similar slight reduction in metabolic activity was observed in splenocytes that were previously activated with the TLR7-agonist R848 at high concentrations of HMPSNE (Fig. 5A, right panel). However, HMPSNE at 50 μM did not significantly affect viability of immune cells (Fig. 5B and Fig. S6A). Furthermore, HMPSNE enhanced T-cell activation within splenocytes activated by R848, as measured by surface expression of the early activation marker CD69. Although no change was seen on total activated splenocytes (defined as CD45^+^ cells), a significant increase in CD69 mean fluorescence intensity was observed in total CD3^+^ T cells, in CD4^+^ helper T cells, and in CD8^+^ cytotoxic T cells at 300 μM HMPSNE (Fig. 5C and Fig. S6B).

The effect of 3-MST inhibition on dendritic cells was also examined. Dendritic cells are antigen-presenting cells that play an essential role in the initiation and orchestration of antitumor immune responses [40]. Dendritic cells were differentiated from mouse bone marrow (bone marrow-derived dendritic cells, BMDC) and exposed to HMPSNE for up to 48 h. No decrease in the percentage of metabolically active BMDCs was observed, even at the highest concentrations of the inhibitor (Fig. 5D). These data suggest that while high concentrations of HMPSNE may modestly affect viability of some immune cell subtypes, the compound also enhances T-cell activation in both the CD4^+^ and CD8^+^ subsets, supporting a potential role for 3-MST in modulating antitumor immune responses.

### 3.7 High concentrations of HMPSNE impair CD8^+^ T cell proliferation without affecting their activation

**Fig. 6:**
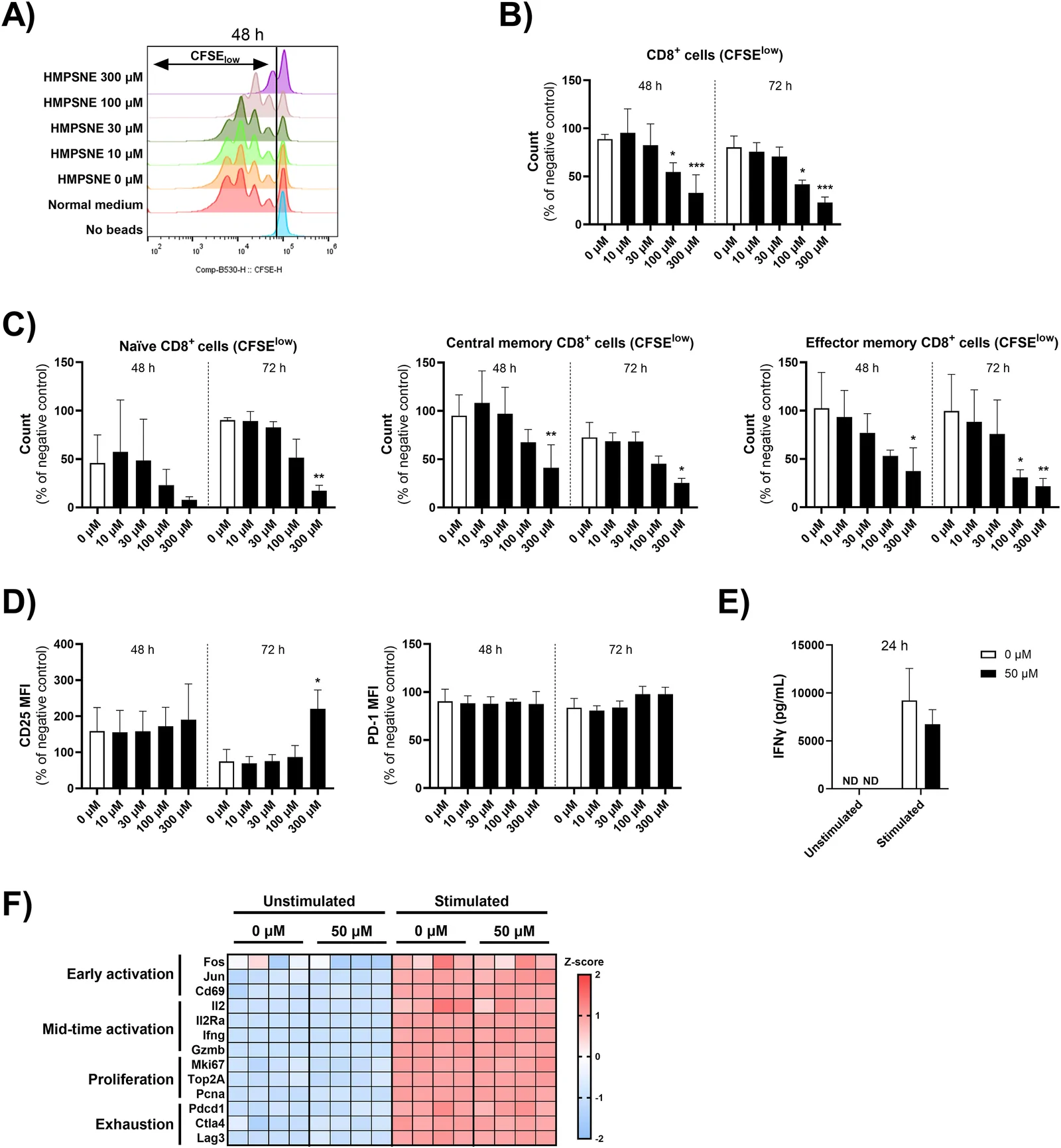
**Effect of HMPSNE on phenotype and proliferation of CD8^+^ T cells.** (**A-D**) CD8^+^ T cells were labelled with CFSE, activated with Dynabeads (anti-CD3/CD28 beads) and IL-2, and treated with various concentrations of HMPSNE for 24, 48 or 72 h. (**A**) Representative CFSE histograms showing cell division peaks after 48 h. (**B**) Quantification of CFSE^low^ CD8^+^ T-cells. (**C**) Quantification of CFSE^low^ CD8^+^ T-cell subsets, including naïve (CD62L^hi^/CD44^lo^), central memory (CD62L^hi^/CD44^hi^), and effector memory (CD62L^lo^/CD44^hi^) populations. (**D**) CD25 and PD-1 expression on CFSE^low^ CD8^+^ T cells (MFI: mean fluorescence intensity). (**B-D**) Data represent the mean ± SD from three independent experiments and are normalized to negative control (untreated cells). Statistical analysis was performed using two-way ANOVA with Dunnett's multiple comparisons test. ***p < 0.001; **p < 0.01; *p < 0.05 vs. vehicle control. (**E-F**) CD8^+^ T cells were activated with Dynabeads (anti-CD3/CD28 beads) and IL-2 and treated with 50 μM of HMPSNE or vehicle for 24 h. (**E**) Quantification of IFNγ production measured by ELISA. Data represent the mean ± SD from at least four independent experiments. (**F**) Heatmap showing the gene expression levels of T cell activation markers, analyzed by RNA-sequencing. Data represent TPM values of four replicates and are displayed as Z-scores.

To assess how inhibition of 3-MST influences CD8^+^ T cell proliferation and phenotype, purified CD8^+^ T cells were labelled with CFSE and stimulated with anti-CD3/CD28 beads and IL-2 in the presence of HMPSNE. Flow cytometric analysis of CFSE dilution revealed that proliferation was unaffected at lower concentrations of HMPSNE (10–30 μM), but reduced at 100 and 300 μM HMPSNE, as evidenced by fewer CFSE^low^ cells (Fig. 6A and B, and Figure S7). This inhibitory effect extended to all examined CD8^+^ T cell subsets, including naïve (CD62L^hi^/CD44^lo^), central memory (CD62L^hi^/CD44^hi^) and effector memory (CD62L^lo^/CD44^hi^) populations, which showed decreased expansion at higher HMPSNE concentrations (Fig. 6C). Despite the reduced proliferation, expression of the activation markers CD25 and PD-1 was maintained (Fig. 6D), and production of the pro-inflammatory cytokine IFNγ was not significantly affected (Fig. 6E). These results suggest that while high dose of HMPSNE can impair CD8^+^ T cell proliferation, it does not suppress activation, highlighting a concentration-dependent effect of 3-MST inhibition on T-cell function.

Transcriptomic analysis further confirmed that 50 μM HMPSNE does not impair CD8^+^ T-cell activation, as shown by the comparable expression of activation-associated genes in HMPSNE- and vehicle control-treated cells (Fig. 6F). Principal component analysis (PCA) showed that CD8^+^ T cells clustered primarily according to their activation status rather than HMPSNE treatment, forming two distinct clusters corresponding to unstimulated and stimulated cells (Fig. S8A). Accordingly, very few genes were differentially expressed between HMPSNE- and vehicle-treated CD8^+^ T cells, particularly compared with Renca cells (Fig. S8B). Interestingly, 19 HMPSNE-induced differentially expressed genes were similarly modulated in Renca cells and CD8^+^ T cells: in both cell types, upregulated genes were predominantly involved in metabolic pathways, whereas downregulated genes included genes associated with oncogenic processes and T-cell exhaustion (Fig. S8C). Collectively, these findings indicate that HMPSNE has only a limited effect on the transcriptional profile of CD8^+^ T cells and does not compromise their activation, suggesting that 3-MST inhibition may impair renal cancer progression without suppressing antitumor T-cell responses.

### 3.8 3-MST inhibition enhances CD8^+^ T cell-mediated cytotoxicity and cooperates with PD-L1 blockade

**Fig. 7:**
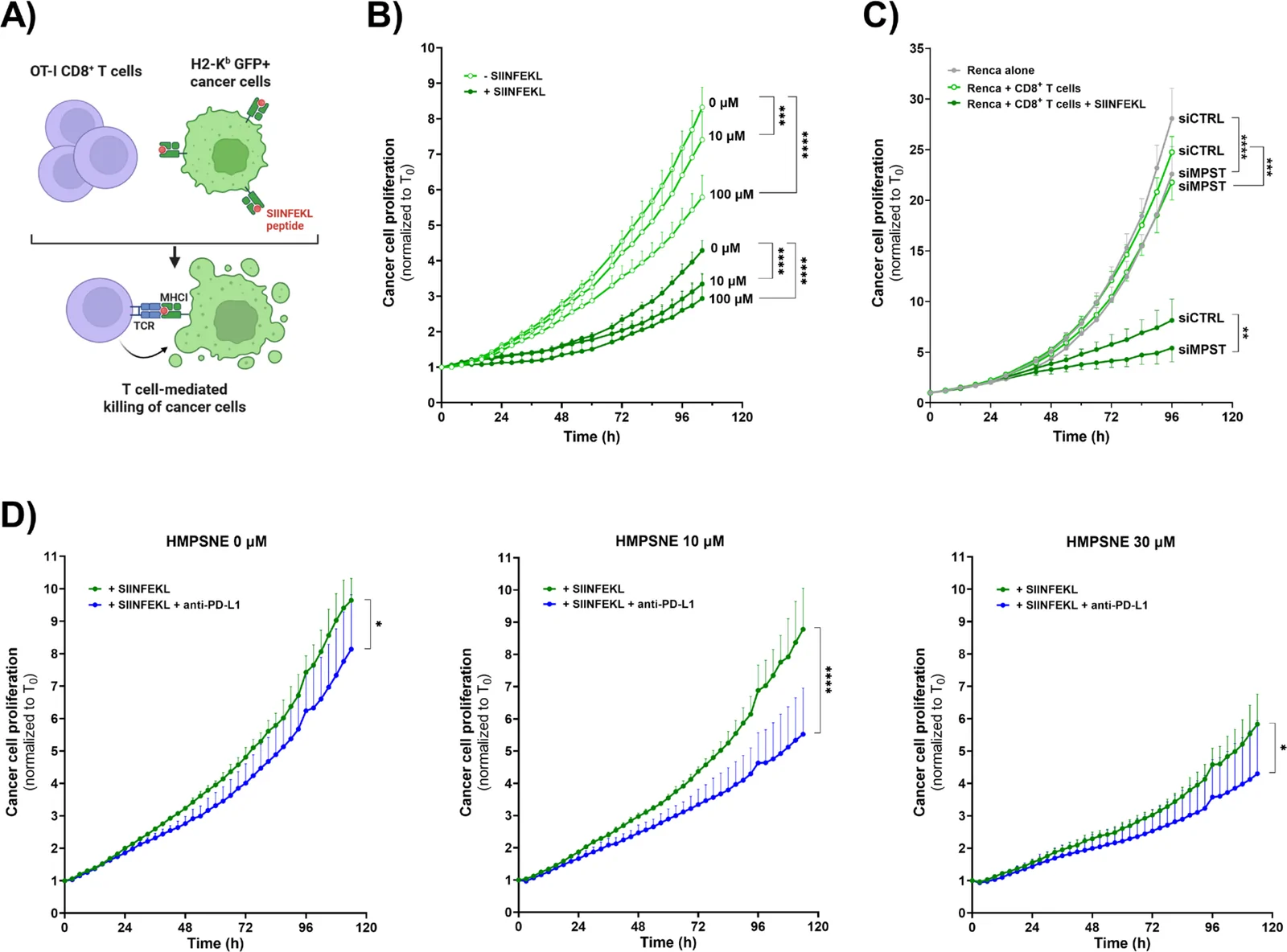
**Impact of 3-MST inhibition on CD8^+^ T cell-mediated cytotoxicity.** CD8^+^ T cell-mediated cytotoxicity was assessed using Renca-H2-Kb GFP^+^ cancer cells either loaded with SIINFEKL or left unloaded, and co-cultured with CD8^+^ T cells isolated from OT-1 mice. (**A**) Schematic representation of the cytotoxicity assay. (**B-C**) Proliferation curves of cancer cells loaded or not with SIINFEKL, co-cultured with CD8^+^ T cells (**B**) in the presence of increasing concentrations of HMPSNE or (**C**) after treatment with *Mpst*-targeting siRNA (siMPST) or non-targeting siRNA (siCTRL). (**D**) Proliferation of cancer cells treated with various concentrations of HMPSNE and/or anti-PD-L1 and co-cultured with CD8^+^ T cells. (**B-D**) Data represent GFP signal normalized the starting point (T_0_ = 1) for each well, and are depicted as mean ± SD from one experiment with three replicates, representative of at least two independent experiments. Statistical analysis was performed using two-way ANOVA with (A) Dunnett's or (B, C) Tukey's multiple comparisons test. ****p < 0.0001; ***p < 0.001; **p < 0.01; *p < 0.05 vs. indicated sample.

To evaluate whether 3-MST inhibition affects antigen-specific T cell-mediated killing of cancer cells, we co-cultured SIINFEKL-loaded GFP^+^ Renca-H2-Kb target cells with CD8^+^ T cells isolated from OT-I mice in the presence or absence of HMPSNE (Fig. 7A). SIINFEKL is an immunodominant peptide epitope from ovalbumin that is specifically recognized by CD8^+^ T cells from OT-I transgenic mice. GFP-based proliferation tracking revealed that cancer cell growth was reduced when cells were loaded with SIINFEKL peptide in the presence of OT-I CD8^+^ T cells, and that this effect was further potentiated by HMPSNE in a dose-dependent manner (Fig. 7B). In the absence of SIINFEKL loading, OT-I cells exerted only a modest inhibitory effect on cancer cell growth, consistent with limited bystander killing, but HMPSNE treatment further enhanced this inhibitory effect. Silencing of the *Mpst* gene replicated the inhibitory effect of HMPSNE treatment, with siMPST Renca cells exhibiting a reduced proliferation capacity compared to cells treated with non-targeting siRNA (Fig. 7C).

We next tested whether immune checkpoint inhibition further amplifies T cell-mediated cytotoxicity. Addition of anti-PD-L1 antibody significantly enhanced OT-I-mediated cytotoxicity against SIINFEKL-loaded target cells (Fig. 7D, left panel), whereas anti-PD-L1 alone had no effect in the absence of SIINFEKL (data not shown), indicating that checkpoint blockade requires effective antigen recognition to exert its activity. Importantly, combining anti-PD-L1 with partial inhibition of 3-MST activity––achieved by low concentrations of HMPSNE––further suppressed cancer cell proliferation compared to checkpoint inhibition alone. These findings suggest that 3-MST inhibition can enhance CD8^+^ T cell effector function and sensitize Renca cancer cells to immune checkpoint blockade, potentially by increasing their immunogenicity and/or altering redox-dependent survival pathways.

## 4 Discussion

H_2_S is recognized as a pivotal gasotransmitter in redox biology, exerting either cytoprotective or cytotoxic effects depending on its concentration, cellular context, and disease state [[41], [42], [43]]. Within the tumor microenvironment, dysregulated H_2_S metabolism is increasingly recognized as a driver of malignant progression, metabolic reprogramming, and immune escape [44]. Among the three main H_2_S-synthesizing enzymes, CBS, CSE, and 3-MST, our analysis of publicly available datasets identified 3-MST as the most abundantly expressed at the protein level in both healthy kidney tissue and RCC. This pattern is consistent with previous reports of elevated 3-MST in other malignancies, including colon adenocarcinoma, lung adenocarcinoma, and urothelial carcinoma [14]. We further show that high 3-MST expression is associated with reduced survival in RCC patients, implicating this enzyme and its product, H_2_S, in disease progression.

Pharmacological inhibition of 3-MST with HMPSNE led to a dose-dependent reduction in intracellular H_2_S levels in Renca cells, consistent with prior reports of HMPSNE specificity [25]. This inhibition suppressed proliferation and induced apoptosis, reinforcing earlier observations that endogenous H_2_S protects cancer cells from oxidative stress and apoptosis through multiple mechanisms [8,45]. Given the established role of H_2_S in redox regulation, its production is expected to be coupled to mitochondrial metabolism. Consistent with this, we found 3-MST to be primarily localized to the mitochondria in renal cancer cells. Interestingly, 3-MST blockade also triggered immunogenic remodeling of tumor cells, with increased expression of co-stimulatory molecules (CD70, CD86), as well as enrichment of genes involved in key immune signaling pathways. These changes mirror redox-driven immune phenotypes described previously, where reactive oxygen species (ROS) enhance antigen presentation machinery and checkpoint expression [46,47]. This immunogenic shift may render tumor cells more susceptible to immune recognition and therapeutic intervention, especially in the context of checkpoint blockade.

While these promising findings suggest a tumor-promoting role for 3-MST-mediated H_2_S production, several limitations should be acknowledged. The TCGA-KIRC cohort analysis did not account for competing risk events, and validation in a prospective patient cohort is necessary. Moreover, our findings will need to be validated in *in vivo* models, as cell culture conditions do not fully replicate the complexity of the tumor microenvironment. Importantly, the *in vivo* efficacy and safety of HMPSNE will also depend on its systemic metabolism, pharmacokinetic properties and potential off-target effects. Finally, it will be important to determine whether these findings extend to other types of cancer, given the broad expression of 3-MST across tissues and tumors.

A striking aspect of our findings is the differential sensitivity of cancer cells versus immune cells to 3-MST inhibition. Whereas Renca cells showed a marked loss of viability, splenocytes and dendritic cells were comparatively resistant to the cytotoxic effects of HMPSNE. This difference may be partly explained by differential *Mpst* expression, which was nearly threefold higher in Renca cells than in CD8^+^ T cells (data not shown). However, differences in *Mpst* expression are unlikely to provide the sole explanation, as HMPSNE elicited distinct effects on immune cells. Specifically, HMPSNE did not alter their metabolic activity and appeared to enhance lymphocyte activation, as evidenced by increased CD69 expression on both CD4^+^ and CD8^+^ T cells following Toll-like receptor (TLR) stimulation. CD69, an early activation marker, can be induced via TLR or cytokine signals independent of antigen-specific T cell receptor engagement [40]. This suggests that modulating the reactive sulfur homeostasis via 3-MST inhibition may preferentially promote effector immune pathways over regulatory or tolerogenic programs. However, at higher HMPSNE concentrations, CD8^+^ T cell proliferation was impaired despite preserved activation marker expression and IFNγ secretion, suggesting a dose-dependent threshold in redox homeostasis—a concept consistent with the role of H_2_S as a redox rheostat, where both depletion and excess can compromise immune function [5,10].

The most compelling translational insight from this study is the cooperative effect between 3-MST inhibition and CD8^+^ T cell-mediated cytotoxicity. Pharmacological inhibition of 3-MST promoted the cytotoxic activity of CD8^+^ T cells against antigen-specific cancer cell targets and further enhanced efficacy when combined with PD-L1 blockade. This cooperative effect suggests that H_2_S-targeting metabolic interventions can sensitize cancer cells to immune attack, possibly by boosting immune costimulatory signals, altering redox-sensitive survival pathways, or reducing metabolic competition at the tumor–immune interface. While further studies are needed to validate these findings using *in vivo* preclinical studies and human RCC models, our data support a model in which 3-MST sustains a tumor-permissive redox state that limits immune activation and promotes cancer cell survival. Targeting this pathway may represent a therapeutic strategy to simultaneously disrupt tumor metabolism and reawaken antitumor immunity.

## CRediT authorship contribution statement

**Muriel Urwyler:** Investigation. **Marietta Margareta Korsos:** Investigation, Writing – original draft. **Eloise Dupuychaffray:** Investigation, Writing – original draft. **Nikita Markov:** Investigation. **Kelly Ascenção:** Investigation. **Maria Petrosino:** Investigation. **Karim Zuhra:** Investigation. **Darko Stojkov:** Investigation. **Sara Barcos:** Investigation. **Jérémie Tachet:** Investigation. **Pragallabh Purwar:** Investigation. **Montserrat Alvarez:** Investigation. **Aurélien Pommier:** Supervision. **Mohamed Z. Gad:** Supervision. **Reham M. Abdel-Kader:** Supervision. **Csaba Szabo:** Conceptualization, Supervision, Funding acquisition. **Carole Bourquin:** Conceptualization, Funding acquisition, Writing – original draft, Writing – review & editing, Project administration, Supervision.

## Declaration of competing interest

The authors declare that they have no known competing financial interests or personal relationships that could have appeared to influence the work reported in this paper.
