# Supplementary material for: Inhibition of 3-mercaptopyruvate sulfurtransferase enhances CD8+ T-cell antitumor immunity
Source: Redox Biol. 2026 Aug 28;97:104373. doi: 10.1016/j.redox.2026.104373 (PMC13570292; doi:10.1016/j.redox.2026.104373)
Supplement: Supplementary file 1 [file mmc1.pdf]

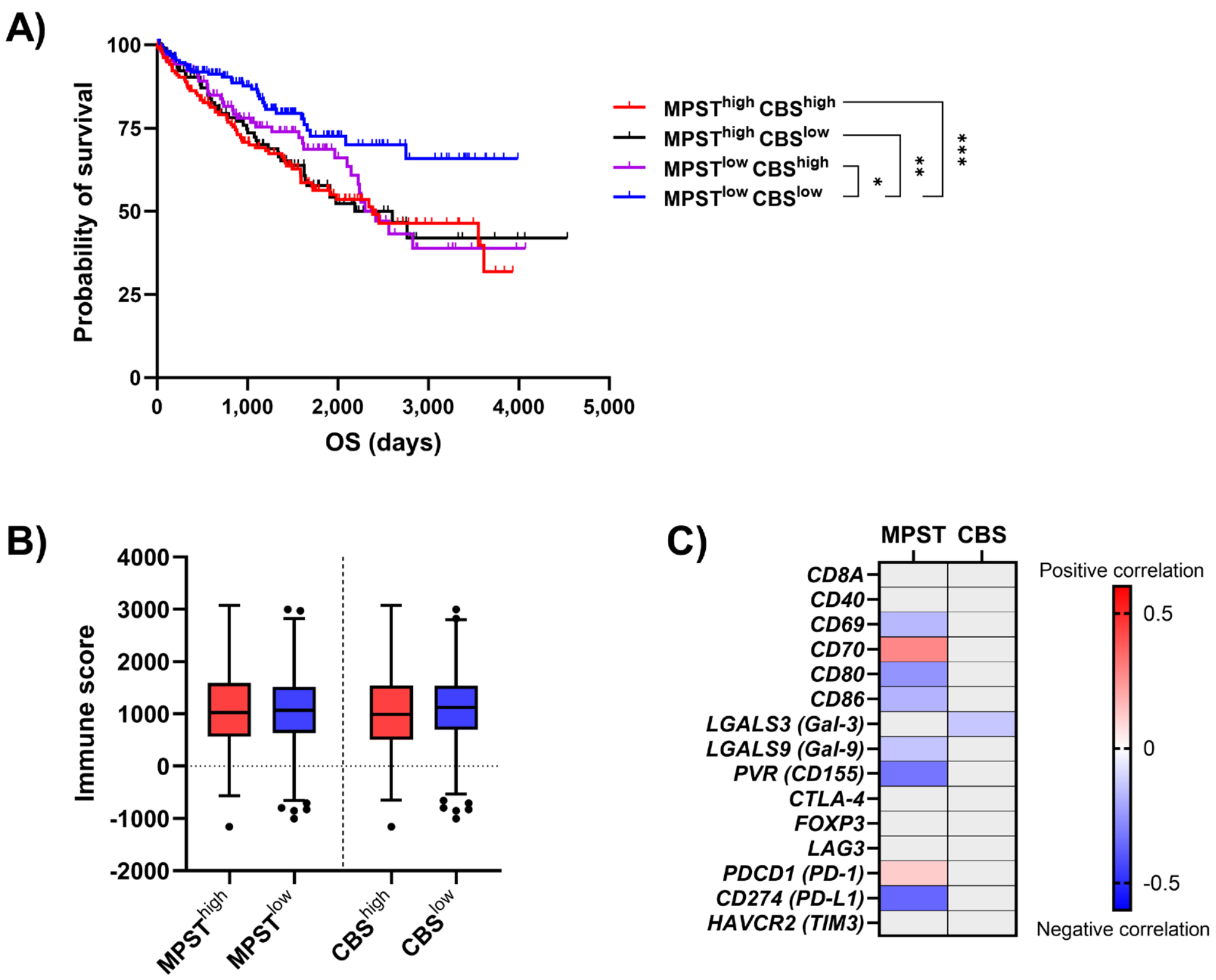


**Figure S1. Correlation between $H_2S$-expressing enzymes, survival of RCC patients, and immune infiltration.** (**A**) Representative images of immunohistochemistry staining of human kidney (left) and human renal cell adenocarcinoma (right) samples from the Human Protein Atlas showing expression of the 3-MST protein. (**B**) Kaplan–Meier curves showing the overall survival (OS) for RCC patients stratified by high vs. low mRNA expression levels of CBS and MPST. Patients were dichotomized into high vs. low gene expression groups based on the median expression values as threshold. Data derived from The Cancer Genome Atlas (TCGA) Kidney Renal Clear Cell Carcinoma (KIRC) cohort (n=533; $MPST^{high}$ $CBS^{high}$ group: n=159, $MPST^{high}$ $CBS^{low}$ group: n=107, $MPST^{low}$ $CBS^{high}$ group: n=107, $MPST^{low}$ $CBS^{low}$ group: n=160). Mantel-Cox test p-values are shown: ***$p < 0.001$; **$p < 0.01$; *$p < 0.05$. (**C**) Immune infiltration in RCC samples calculated with the ESTIMATE algorithm. Data are derived from the TCGA-KIRC cohort (n=533) and are depicted as Tukey's box plot. (**D**) Correlation between the expression of MPST or CBS with several immune-related genes calculated with the TIMER2.0 web platform. Spearman's rho values are shown (red: positive correlation; blue: negative correlation; grey: not significant). Data derived from the TCGA-KIRC cohort (n=533).

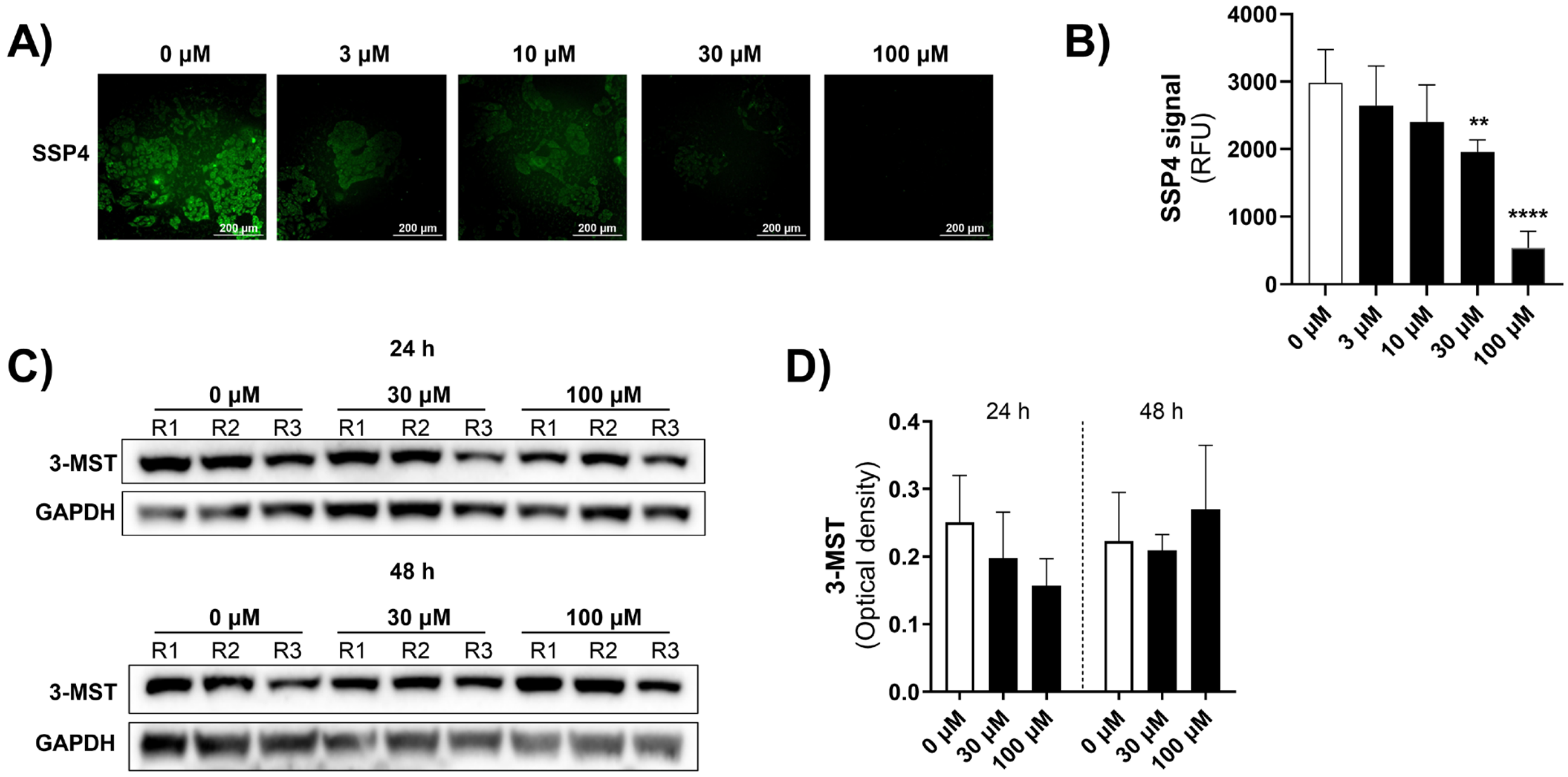


**Figure S2. Impact of HMPSNE on polysulfide production and 3-MST expression.** (**A-B**) Polysulfide content in Renca cells treated with increasing concentrations of HMPSNE for 48 h, quantified using the SSP4 probe. (**A**) Representative SSP4 fluorescence images of cells. Scale bars: 200 µm. (**B**) Quantification of SSP4 fluorescence signal across HMPSNE concentrations. Data represent the mean ± SD. Statistical analysis was performed using one-way ANOVA with Bonferroni's multiple comparisons test. ****p < 0.0001; **p < 0.01 vs. vehicle control. (**C-D**) Renca cells were treated with increasing concentrations of HMPSNE for 24 or 48 h, and cell lysates were analyzed by Western blot, probed with 3-MST (Mw:33kDa) and GAPDH (Mw:36kDa) antibodies. (**C**) Representative immunoblots. (**D**) Quantification of 3-MST protein abundance, normalized to GAPDH. Data represent mean ± SD with three biological replicates.

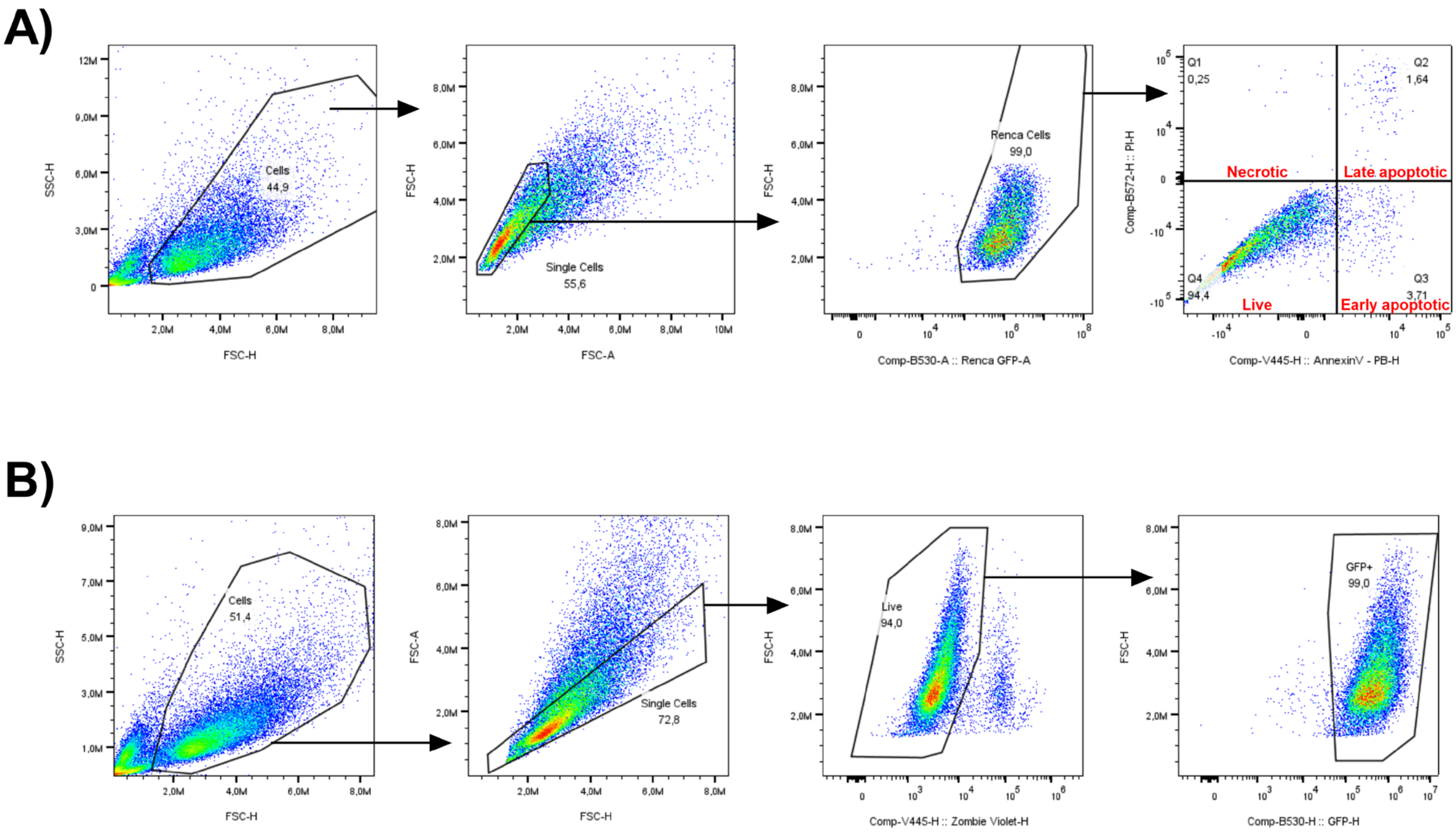


**Figure S3. Gating strategy for analysis of Renca cells.** (**A**) Analysis of cell apoptosis. The first two gates were set to exclude cell debris and select single cells. Then, $GFP^+$ cells were selected and, using propidium iodide (PI) and Annexin V staining, live, early/late apoptotic and necrotic cells were identified. (**B**) Analysis of cell surface phenotyping. The first two gates were set to exclude cell debris and select single cells. Then, a viability marker was used to exclude dead cells. $GFP^+$ cells were selected and mean fluorescence intensity of several cell surface markers was quantified.

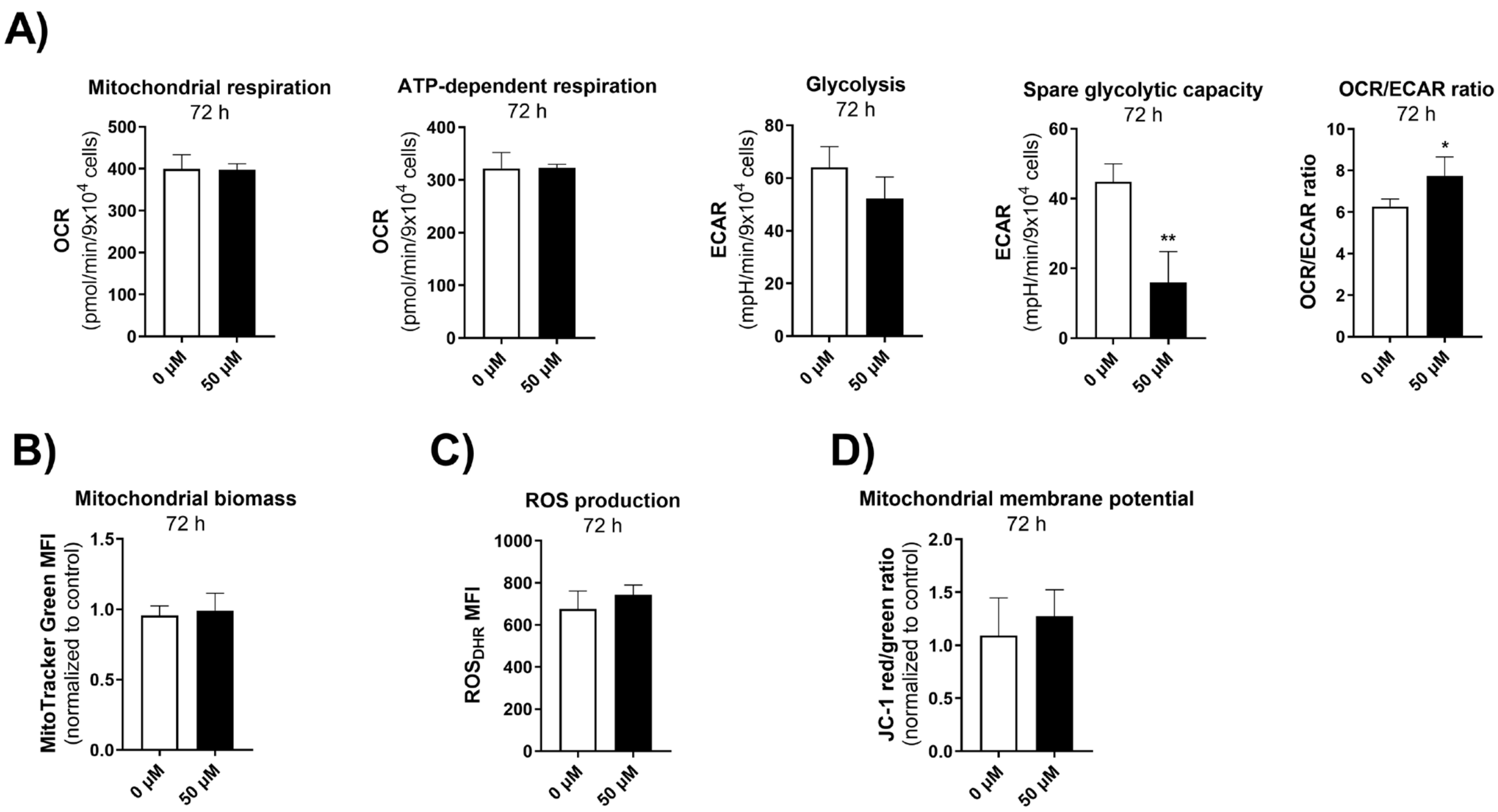


**Figure S4. Effect of HMPSNE on renal cancer cell bioenergetics.** Renca cells were treated with HMPSNE (50 µM) or vehicle control for 72 h. (**A**) Bioenergetic profiling measured by extracellular flux analysis (OCR: Oxygen Consumption Rate; ECAR: Extracellular Acidification Rate). (**B**) Mitochondrial biomass measured by flow cytometry using MitoTracker™ Green FM dye. (**C**) Production of reactive oxygen species (ROS) measured by flow cytometry using dihydrorhodamine 123 dye. (**D**) Mitochondrial membrane potential measured by flow cytometry using JC-1 dye, expressed as the ratio of red fluorescence (JC-1 aggregates) to green fluorescence (JC-1 monomers). Data represent mean ± SD with at least three biological replicates. Statistical analysis was performed using two-way ANOVA with Šidák's multiple comparisons test. ****$p < 0.0001$; **$p < 0.01$; *$p < 0.05$ vs. vehicle control.

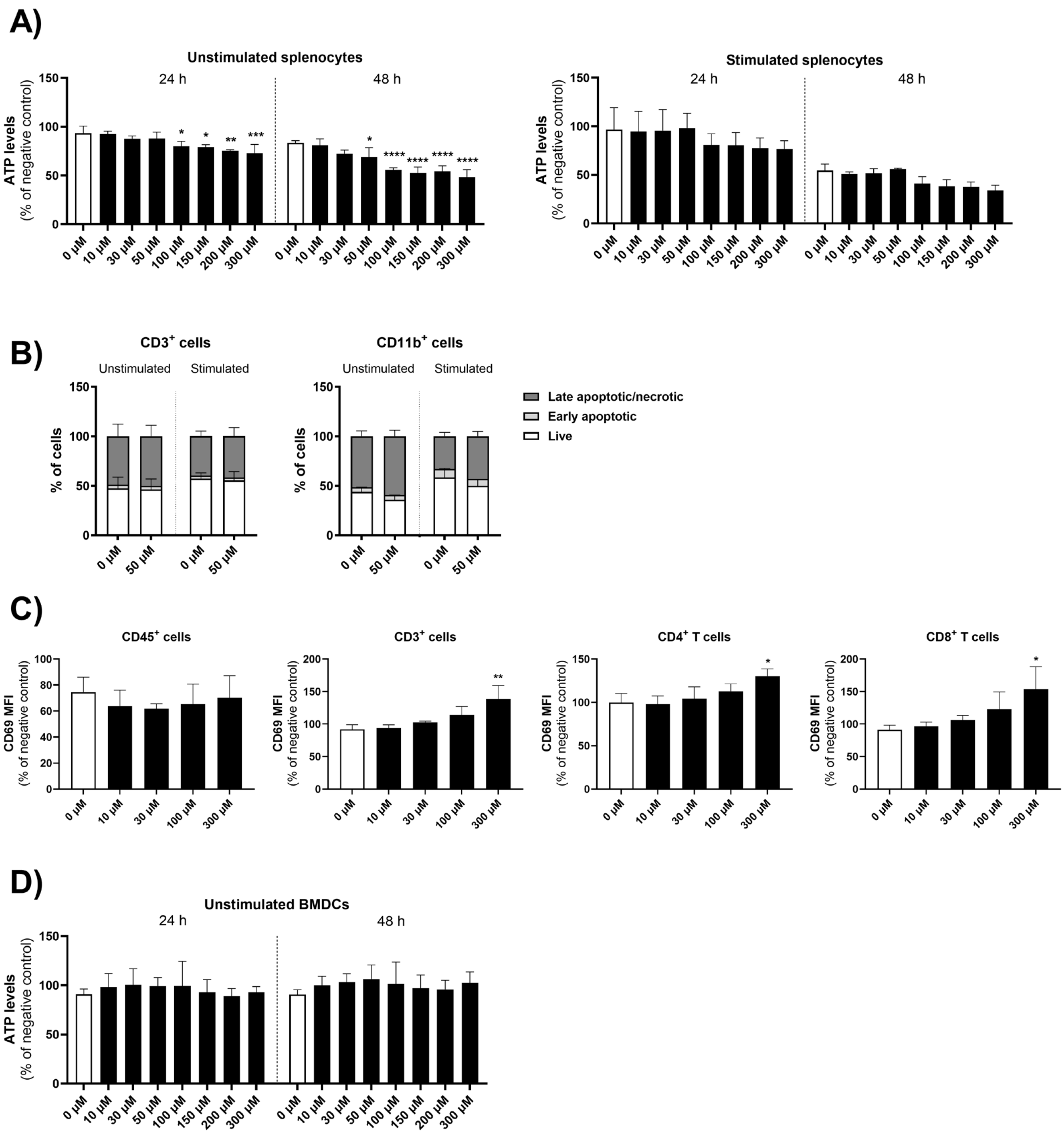


**Figure S5. Effect of *Mpst*-silencing in renal cancer cells.** Renca cells were treated with siRNA targeting *Mpst* (siMPST, 20 pmol) for 48 h, followed by a 24-hour recovery period after media replacement. Untreated cells, cells treated without siRNA (Reagent), and cells treated with non-targeting siRNA (siCTRL) were used as controls. (**A-B**) Representative immunoblots of cell lysates, probed with 3-MST (Mw:33kDa) and Lamin A (Mw:70kDa) antibodies, showing the optimization of the *Mpst* silencing protocol. The selected conditions for further experiments are outlined. (**C**) *Mpst* expression after silencing, measured by qPCR. (**D**) Quantification of live cells after silencing. (**E-F**) Cellular $H_2S$ and polysulfide content after silencing, quantified using the AzMC probe or the SSP4 probe, respectively. (**E**) Quantification of cellular $H_2S$ (left) and polysulfide (right) content. (**F**) Representative AzMC fluorescence (top) and SSP4 fluorescence (bottom) images of cells. Scale bars: 200 µm. (**G**) Bioenergetic profiling measured by extracellular flux analysis (OCR: Oxygen Consumption Rate; ECAR: Extracellular Acidification Rate). (**H**) Mitochondrial biomass measured by flow cytometry using MitoTracker™ Green FM dye. (**I**) Production of reactive oxygen species (ROS) measured by flow cytometry using dihydrorhodamine 123 dye. (**J**) Mitochondrial membrane potential measured by flow cytometry using JC-1 dye, expressed as the ratio of red fluorescence (JC-1 aggregates) to green fluorescence (JC-1 monomers). (**C**, **D** and **G-**

**J**) Data represent mean ± SD with at least three biological replicates. Statistical analysis was performed using one-way ANOVA with Šidák's (C, D) or Bonferroni's (E) multiple comparisons test, or using paired t-test (G-J). ****$p < 0.0001$; ***$p < 0.001$; **$p < 0.01$; *$p < 0.05$ vs. siCTRL. (**K-L**) The gene expression profile was analyzed by RNA-sequencing. (**K**) Enrichment analysis on the differentially expressed genes (DEG) using the KEGG database. (**L**) Heatmap of the DEG from the "Antigen processing and presentation" signature. Data represent TPM values of four replicates and are displayed as Z-scores.

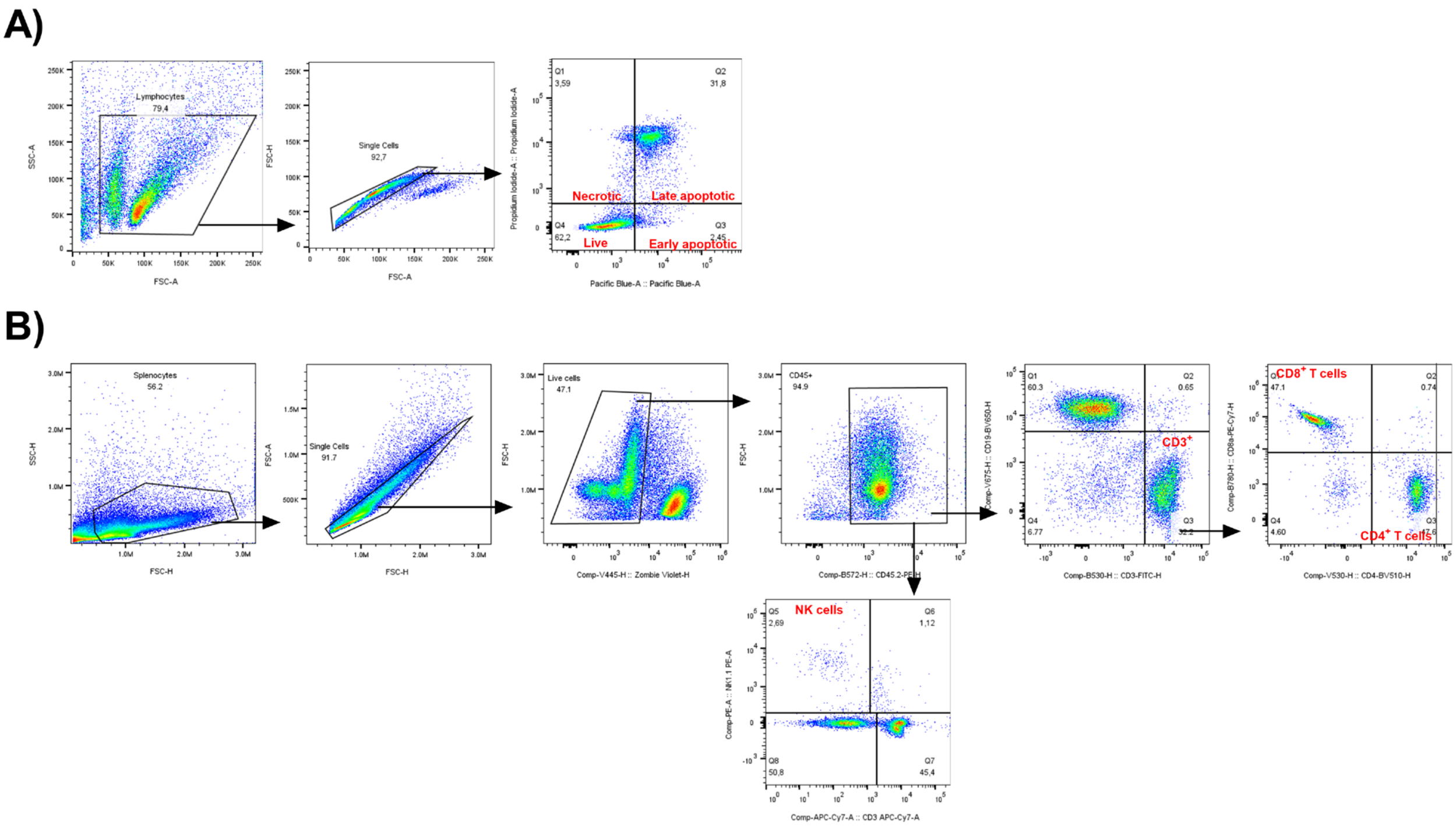


**Figure S6. Gating strategy for analysis of splenocytes.** (**A**) Analysis of cell apoptosis. The first two gates were set to exclude cell debris and select single cells. Then, using propidium iodide (PI) and Annexin V staining, live, early/late apoptotic and necrotic cells were identified. Similar gating strategy was used on total T cells, $CD4^+$ T cells, $CD8^+$ T cells, and NK cells. (**B**) Analysis of cell populations. The first two gates were set to exclude cell debris and select single cells. Then, a viability marker was used to exclude dead cells. The gate on the $CD45.2^+$ population ensured the inclusion of splenocytes only. Then, different subsets of splenocytes were defined as follows: total T cells ($CD3^+$ $CD19^-$), $CD4^+$ T cells ($CD3^+$ $CD4^+$), $CD8^+$ T cells ($CD3^+$ $CD8^+$), NK cells ($NK1.1^+$ $CD3^-$).

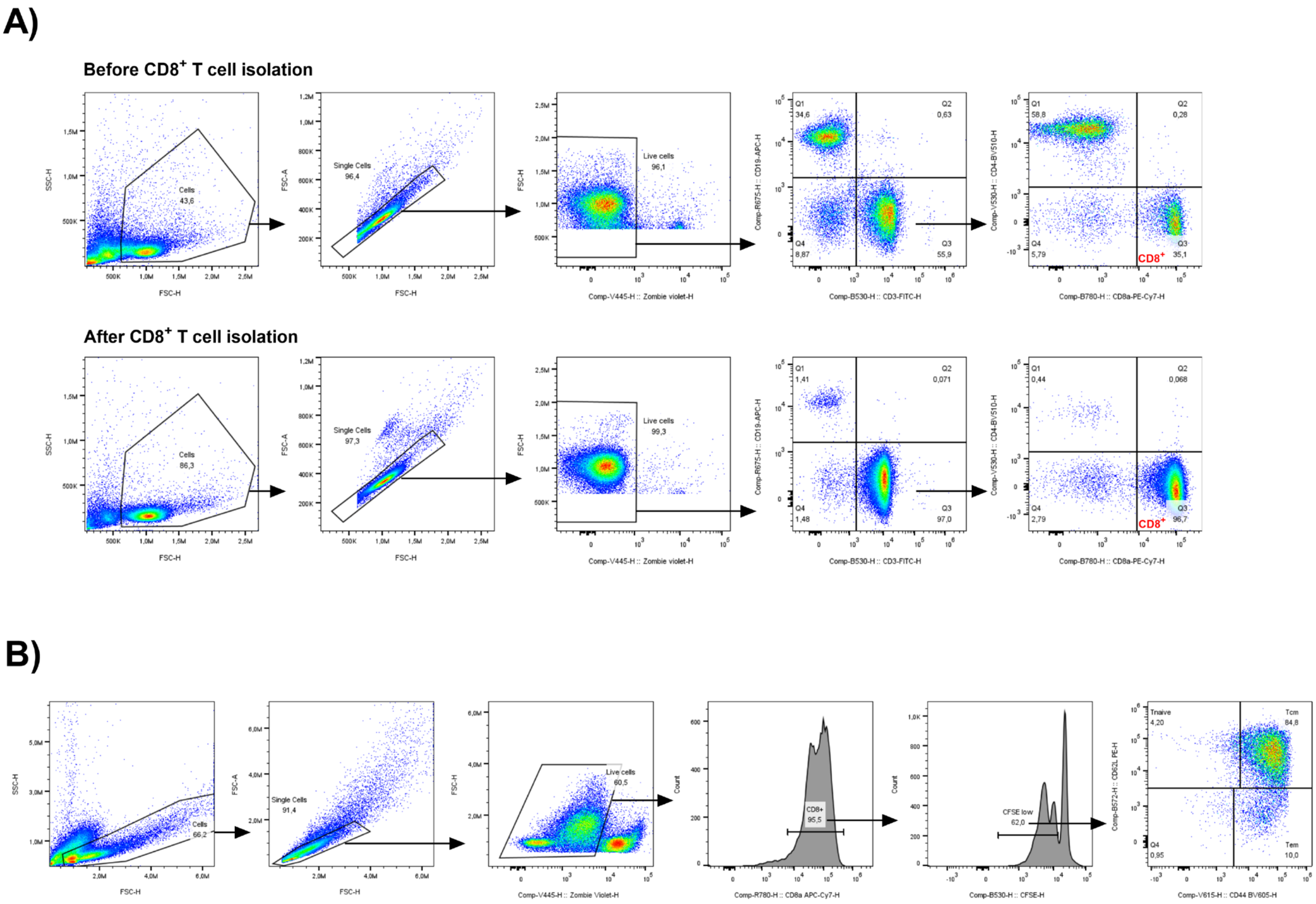


**Figure S7. Gating strategy for analysis of $CD8^+$ T cells.** (**A**) Analysis of cell purity before (top) and after (bottom) isolation of $CD8^+$ T cells from mouse splenocytes. The first two gates were set to exclude cell debris and select single cells. Then, a viability marker was used to exclude dead cells. The gate on the $CD3^+$ population ensured the inclusion of T cells only. Then, $CD8^+$ T cells were defined as $CD3^+$ $CD8^+$ cells. (**B**) Analysis of proliferating $CD8^+$ T cells. The first two gates were set to exclude cell debris and select single cells. Then, a viability marker was used to exclude dead cells. The gate on the $CD8^+$ population ensured the purity of the cell population. Then, proliferating $CD8^+$ T cells were identified as $CFSE^{low}$ cells, and different subsets of $CD8^+$ T cells were defined as follows: naive ($CD44^{low}$ $CD62L^{high}$), effector memory (Tem; $CD44^{high}$ $CD62L^{low}$), central memory (Tcm; $CD44^{high}$ $CD62L^{high}$).

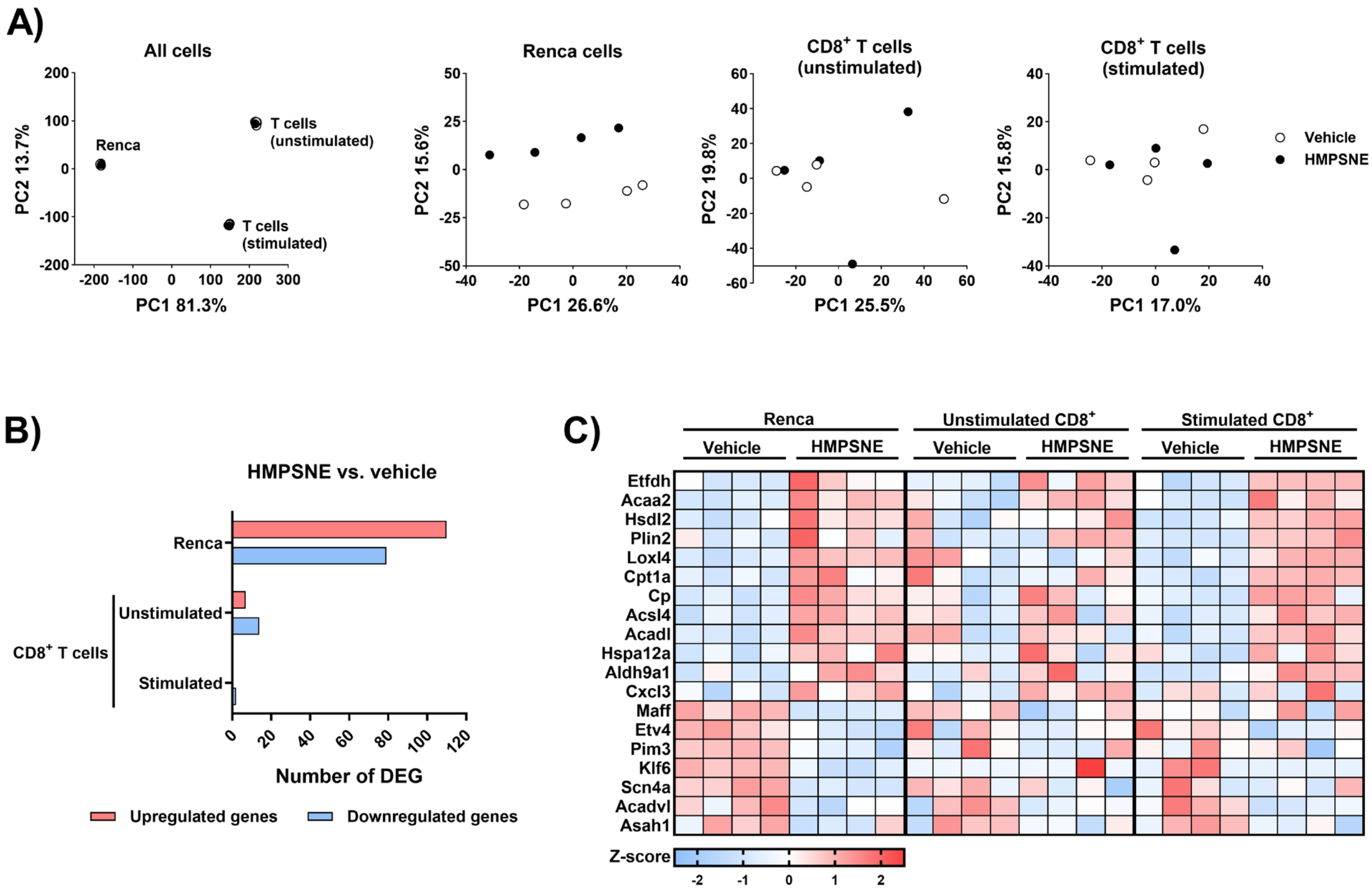


**Figure S8. Comparison of HMPSNE transcriptional effects on CD8$^+$ T cells and Renca cells.** Renca-H2-Kb GFP$^+$ cells were treated with HMPSNE (50 µM) or vehicle control for 72 h. CD8$^+$ T cells were either left unstimulated or activated with Dynabeads (anti-CD3/CD28 beads), and treated with HMPSNE (50 µM) or vehicle control for 24 h. Gene expression profile was analyzed by RNA-sequencing (n=4 per group). (**A**) 3D principal component analysis (PCA) clustering. (**B**) Number of differentially expressed genes (DEG) in HMPSNE-treated cells vs. vehicle-treated cells. (**C**) Heatmap of the common DEG between HMPSNE-treated cells and vehicle-treated cells in Renca, unstimulated CD8$^+$ T cells, and stimulated CD8$^+$ T cells.

**Supplementary Table S1. FACS panels and amine-reactive dyes**

| Panel | Antibody | Fluorochrome | Dilution | Provider | Ref. number |
|---|---|---|---|---|---|
| **Renca-H2-Kb GFP⁺ immunophenotyping** | anti-mouse CD16/32 | - | 1/100 | Biolegend | 101319 |
| | anti-CD86 | BV510 | 1/200 | Biolegend | 105040 |
| | anti-CD70 | APC | 1/200 | Biolegend | 104610 |
| | anti-PD-L1 | BV785 | 1/200 | Biolegend | 124331 |
| | anti-CD155 | PE | 1/200 | Biolegend | 131508 |
| | anti-Galectin 9 | PE/Cy7 | 1/200 | Biolegend | 137914 |
| | anti-MHC I | PerCP/Cy5.5 | 1/200 | Biolegend | 116515 |
| **Mouse T cell purity** | anti-mouse CD16/32 | - | 1/100 | Biolegend | 101319 |
| | anti-CD3 | FITC | 1/200 | Biolegend | 100203 |
| | anti-CD8a | PE | 1/200 | Biolegend | 100708 |
| | anti-CD4 | BV510 | 1/200 | Biolegend | 100559 |
| | anti-CD19 | APC | 1/200 | Biolegend | 115512 |
| **Mouse T cell immunophenotyping from splenocytes** | anti-mouse CD16/32 | - | 1/100 | Biolegend | 101319 |
| | anti-CD45 | PE | 1/200 | MACS | 130-102-498 |
| | anti-CD3 | FITC | 1/200 | Biolegend | 100203 |
| | anti-CD3 | APC-Cy7 | 1/200 | Biolegend | 100222 |
| | anti-CD8a | PE/Cy7 | 1/200 | Biolegend | 100722 |
| | anti-CD69 | APC | 1/200 | Biolegend | 104513 |
| | anti-CD4 | BV510 | 1/200 | Biolegend | 100553 |
| | Anti-NK1.1 | PE | 1/200 | Biolegend | 156504 |
| **Mouse T cell CFSE panel** | anti-mouse CD16/32 | - | 1/100 | Biolegend | 101319 |
| | anti-CD62L | PE | 1/200 | Biolegend | 104408 |
| | anti-PD-1 | PE/Cy7 | 1/200 | Biolegend | 135216 |
| | anti-CD25 | APC | 1/200 | Biolegend | 102012 |
| | anti-CD69 | BV510 | 1/200 | Biolegend | 104532 |
| | anti-CD44 | BV605 | 1/200 | Biolegend | 103047 |
| **Mouse BMDC panel** | anti-CD11c | BV570 | 1/200 | Biolegend | 117331 |
| | anti-CD80 | BV605 | 1/200 | Biolegend | 104729 |
| | anti-PD-L1 | BV785 | 1/200 | Biolegend | 124331 |
| | anti-MHC II | FITC | 1/200 | MACS | 130-123-666 |
| | anti-CD40 | PE | 1/200 | eBioscience | 12-0401-82 |
| | anti-CD11b | PerCP/Cy5.5 | 1/200 | BD | 550993 |
| | anti-MHC I | APC | 1/200 | eBioscience | 17-5958-80 |
| | anti-CD86 | APC/Cy7 | 1/200 | Biolegend | 105030 |
| **Viability dyes** | - | Zombie Violet | - | Biolegend | 423114 |
| | - | Zombie Green | - | Biolegend | 423112 |
| | - | Propidium iodide | 1/1000 | Sigma | P4864 |
| | Annexin V | Pacific Blue | - | Invitrogen | R37177 |
